# When barcoding fails: Genome chimerization (admixing) and reticulation obscure phylogenetic and taxonomic relationships

**DOI:** 10.1111/1755-0998.13586

**Published:** 2022-02-03

**Authors:** Matthias Sipiczki

**Affiliations:** ^1^ Department of Genetics and Applied Microbiology University of Debrecn Debrecen Hungary

**Keywords:** barcodes, *Metschnikowia*, mosaic genome, network analysis, phylogenetic analysis, reticulation, rRNA secondary structure

## Abstract

DNA barcoding is based on the premise that the barcode sequences can distinguish individuals (strains) of different species because their sequence variation between species exceeds that within species. The primary barcodes used in fungal and yeast taxonomy are the ITS segments and the LSU (large subunit) D1/D2 domain of the homogenized multicopy rDNA repeats. The secondary barcodes are conserved segments of protein‐encoding genes, which usually have single copies in haploid genomes. This study shows that the analysis of barcode sequences fails to reconstruct accurate species trees and differentiate species when the organisms have chimeric genomes composed of admixed mosaics of different origins. It is shown that the type strains of 10 species of the *pulcherrima* clade of the ascomycetous yeast genus *Metschnikowia* cannot be differentiated with standard barcodes because their intragenomic diversity is comparable to or even higher than the interstrain diversity. The analysis of a large group of genes of the sequenced genomes of the clade and the viability and segregation of the hybrids of ex‐type strains indicate that the high intragenomic barcode differences can be attributed to admixed genome structures. Because of the mosaic structures of the genomes, the rDNA repeats do not form continuous arrays and thus cannot be homogenized. Since the highly diverse ITS and D1/D2 sequences of the type strains form a continuous pool including pseudogenes, the evolution of their rDNA appears to involve reticulation. The secondary barcode sequences and the nonbarcode genes included in the analysis show incongruent phylogenetic relationships among the type strains, which can also be attributed to differences in the phylogenetic histories of the genes.

## 1 INTRODUCTION

The taxonomic identification of yeasts has been traditionally based on morphological characteristics, but due to their poor morphological diversity, distinctive reactions in a standardized set of fermentation and assimilation tests have also been applied (van der Walt & Yarrow, 1984). However, neither the morphological and physiological traits nor their combinations are adequate for defining taxa and identifying the taxonomic affiliation of strains in all yeast groups. Therefore, systematics has turned increasingly to molecular approaches to distinguish and identify yeast species and to develop a system of classification based on phylogeny (phylogenetic species concept) (Boekhout et al., 2021; Kurtzman et al., 2011).

In classification based on the phylogenetic species concept, the phylogenetic relationships between strains can be investigated by comparison and analysis of so‐called barcodes, sequences of conserved segments of nuclear and mitochondrial genes. Their application is based on the notion that the barcode differences are smaller within the species than between them (Hebert et al., 2003). The difference between the highest intraspecific variation and the smallest interspecific divergence is the barcode gap that separates the species. The D1/D2 domain of the large subunit (LSU) rRNA gene and the internal transcribed spacers ITS1 and ITS2 have become the most widely used barcodes in yeasts (e.g., Fell et al., 2000; Kurtzman & Robnett, 1998; Vu et al., 2016). These fragments are amplified with “universal” primers, and the resulting amplicons are sequenced, routinely with the Sanger method. Where the rDNA barcodes do not suffice to discriminate between species, secondary barcoding markers need to be employed (for reviews, see Lücking et al., 2020; Xu, 2016). The secondary barcodes used in mycology are conserved fragments of certain protein‐coding household genes usually present in single copies in haploid genomes (e.g., actin [*ACT1*], β‐tubulin II [*TUB2*], DNA‐directed RNA polymerase II largest [*RPB1*] and second largest [*RPB2*] subunits, translational elongation factor 1α [*TEF1*], translation elongation factor 2 [*TEF2*/*EF2*] and calmodulin [*CAM*]) (Stielow et al., 2015). However, with the rapidly expanding amount of barcoding results and the increasing number of sequenced genomes, the accuracy and precision of taxon delimitation and taxonomic identification of strains by a handful of barcodes are increasingly being questioned (examples in fungi: Conti et al., 2021; Jiao & Yang, 2021; Lücking et al., 2020; Sipiczki et al., 2013, 2018). Genes different from the “standard barcode genes” can also be used for taxonomic and phylogenetic studies. The *MAT* genes and certain *MAT*‐controlled sexuality genes were successfully applied to the examination of phylogenetic relationships in *Clavispora*, the large‐spored species of *Metschnikowia* (Lee et al. (2018) and *Cryptococcus* (Yurkov et al., 2015).

A caveat to the reliability of rDNA barcoding is that despite repeat homogenization, the multicopy nature of rDNA implies some level of heterogeneity amongst the various repeats. This intragenomic diversity remains undetected in the Sanger sequences as long as the proportion of the divergent (less abundant) repeats is low. It becomes detectable when the presence of the differing nucleotides attains a considerable level at certain sites. At these sites the Sanger sequences have ambiguous nucleotides. The presence of diverse repeats can be demonstrated by cloning and sequencing ITS or D1/D2 fragments of individual rDNA repeats or by assembling complete rDNA arrays from NGS (new generation sequencing) reads. Considerable intragenomic rDNA barcode heterogeneity was detected in a few filamentous fungal species, for example in certain *Amanita*, *Cordyceps*, *Laetiporus* (Hughes et al., 2018; Li et al., 2013; Lindner & Banik, 2011), *Hypoxylon* and *Pyrenopolyporus* (Stadler et al., 2020) strains and, to a much lesser extent, in certain yeasts (e.g., Alper et al., 2011; Chand Dakal et al., 2016; Colabella et al., 2021; Ganley & Kobayashi, 2007; Heeger et al., 2018; Lachance et al., 2003; Roscini et al., 2018; West et al., 2014).

The pulcherrimin‐producing *Metschnikowia* strains represent an example in which the extent of diversity is much higher than in any yeasts for which data are available. Ambiguous nucleotides occur quite frequently in the Sanger sequences of their D1/D2 and ITS barcodes (e.g., Kurtzman & Droby, 2001; Molnar & Prillinger, 2005; Pawlikowska et al., 2019; Sipiczki, 2006; Sipiczki et al., 2013, 2018). Two species (*M*. *andauensis* and *M*. *fructicola*) were even delimited with “unclean” sequences. Detailed analysis of the D1/D2 domains and the ITS sequences revealed that the type strains of these species shared a continuous pool of highly diverse, nonhomogenized and reticulating rDNA repeats with no interrupting interspecies barcode gaps (Sipiczki et al., 2013, 2018). A search for rDNA repeats in the whole‐genome sequence of the *M*. *fructicola* type strain then revealed that its rDNA units are scattered over the entire genome and the strain has both complete and truncate genes (pseudogenes). Both species belong to the “*pulcherrima*” clade of the genus *Metschnikowia* (reviewed in Lachance, 2016; Sipiczki, 2020). The clade consists of 10 species (*M*. *andauensis*, *M*. *citriensis*, *M*. *fructicola*, *M*. *leonuri*, *M*. *persimmonesis*, *M*. *pulcherrima*, *M*. *rubicola*, *M*. *shanxiensis*, *M*. *sinensis* and *M*. *zizyphycola*) (Lachance, 2016), but the taxon names *M*. *citriensis* and *M*. *persimmonesis* are listed in Mycobank as invalid. These yeasts share the ability to produce pulcherrimin, an insoluble complex of ferric ions with pulcherriminic acid secreted by the yeast cells that turns the colonies and the surrounding medium maroon‐red (reviewed in Sipiczki, 2020). Pulcherrima‐clade strains are often used in fermentation technologies and as bioprotective agents (for reviews, see, e.g., Sipiczki, 2020; Vicente et al., 2020). The taxonomic division of the *pulcherrima* clade is based primarily on D1/D2 sequence differences because the testable phenotypic traits are very similar in all species (Lachance, 2011). However, even the sequence‐based division is controversial because the sequences used in the phylogenetic analyses either contained ambiguous nucleotides (Kurtzman & Droby, 2001; Molnar & Prillinger, 2005) or were cloned fragments (Kang et al., 2017; Liu et al., 2018; Xue et al., 2006). The latter only represented individual rDNA repeats. Unfortunately, very few secondary barcodes were sequenced for the *pulcherrima*‐clade yeasts, and some fragments amplified with primers matching secondary barcodes also contained ambiguous nucleotides (Kurtzman et al., 2018; Molnar & Prillinger, 2005). The latter observation indicates that the secondary barcode genes can also be present in more than one copy in certain genomes.

The aim of this study was to use the ex‐type strains of the clade to examine the applicability of barcoding to the differentiation of species whose strains show high intragenomic barcode diversity. The main objectives included (i) investigation of the intragenome D1/D2 and ITS diversity in the type strains of the *pulcherrima* clade by cloning barcode segments of individual rDNA repeats and by examination of the rDNA repeats in sequenced genomes, (ii) revealing which types of substitutions take place (are tolerated by the evolution) in which positions by predicting secondary structures of transcripts, (iii) exploration of the intragenome diversity of secondary barcodes by cloning the relevant genes and scanning genome sequences, and examination of (iv) the involvement of genome chimerization and (v) reticulation in the evolution of the barcodes by phylogenetic and network analyses. The results show that because of the chimeric genome structures and the interspecies reticulation, neither rDNA nor standard secondary barcodes can correctly distinguish the species in the clade. More complex methodological approaches have to be developed for examination of the phylogenetic relationships when genome chimerization and reticulation are involved in the evolution of the strains.

## 2 MATERIALS AND METHODS

### 2.1 Strains and culture conditions

All strains used in the experiments are listed in Table 1. The composition and application of growth media are described in the Methods section of the Supporting Information.

**TABLE 1:** List of strains

Identification number				
In‐house collection	CBS	Taxonomic name/strain name^a^	Phenotype (genetic marker)	Source^b^
Ex‐type cultures				
11‐578	CBS 5833	*M*. *pulcherrima* ^T^	Wild type	CBS
11‐579	CBS 8853	*M*. *fructicola* ^T^	Wild type	CBS
11‐1088	CBS 10357	*M*. *sinensis* ^T^	Wild type	CBS
11‐1089	CBS 10358	*M*. *zizyphicola* ^T^	Wild type	CBS
11‐1090	CBS 10359	*M*. *shanxiensis* ^T^	Wild type	CBS
11‐1120	CBS 10809	*M*. *andauensis* ^T^	Wild type	CBS
Mutants for hybridization				
11‐1591	CBS 15390	*M*. *pulcherrima* Mp5	his^−^ α	Sipiczki et al. (2018)
11‐1592	CBS 15381	*M*. *pulcherrima* Mp12	ade^−^ a	Sipiczki et al. (2018)
11‐1593		*M*. *pulcherrima* Mp13	lys^−^ α	Sipiczki et al. (2018)
11‐1455		*M*. *fructicola* Mf1455	lys^−^ his^−^	This study
11‐1456		*M*. *fructicola* Mf1456	lys^−^ pro^−^	Sipiczki et al. (2018)
11‐1457		*M*. *fructicola* Mf1457	lys^−^ arg^−^	This study
11‐1458		*M*. *fructicola* Mf1458	ade^−^ his^−^	Sipiczki et al. (2018)
11‐1594		*M*. *fructicola* Mf3	pro^−^	This study
11‐1595		*M*. *fructicola* Mf16	aux	This study
11‐1596	CBS 15376	*M*. *andauensis* Ma22	ade^−^	Sipiczki et al. (2018)
11‐1597		*M*. *andauensis* Ma23	ade^−^	This study
11‐1598	CBS 15377	*M*. *andauensis* Ma28	arg^−^	Sipiczki et al. (2018)
11‐1599		*M*. *andauensis* Ma32	try^−^	This study
11‐1609		*M*. *sinensis* Msi40	ade^−^	This study
11‐1610		*M*. *sinensis* Msi41	ade^−^	This study
11‐1612		*M*. *sinensis* Msi43	ade^−^	This study
11‐1604		*M*. *zizyphicola* Mz33	asp^−^	This study
11‐1621		*M*. *zizyphicola* Mz38	lys^−^	This study
11‐1622		*M*. *zizyphicola* Mz45	ade^−^	This study
11‐1605		*M*. *shanxiensis* Msh19	asp^−^	This study
11‐1620		*M*. *shanxiensis* Msh20	his^−^	This study

^a^ T, type strain.

^b^ CBS: CBS‐KNAW Culture Collections (Utrecht, Netherlands).

### 2.2 Molecular methods

DNA was isolated from overnight cultures grown in YEL as described previously (Sipiczki, 2003) and the isolated DNA was used for the amplification, cloning and sequencing of barcodes. The description of the primers and the procedures is provided in the Methods section of the Supporting Information. The sequences were deposited in GenBank under accession numbers listed in Table S1. The clones were compared with each other by aligning each cloned sequence with all other cloned sequences using the EMBOSS distmat algorithm (https://www.bioinformatics.nl/cgi‐bin/emboss/distmat).

### 2.3 Search for sequences in the INSDC databases and in *Metschnikowia* genome sequences

Barcode sequences and sequences of genes coding for conserved proteins were identified by blastn searches and downloaded from the INSDC databases and the Genome database of NCBI as described in the Methods section of the Supporting Information.

### 2.4 Phylogenetic and network analyses

The phylogenetic relationships were investigated by analysis of the sequences with neighbour‐joining (NJ), maximum‐likelihood (ML), statistical parsimony network and neighbour‐net splits graph algorithms as described in the Supporting Information.

### 2.5 RNA secondary structure prediction

In a previous study, we observed that the variable positions of the D1/D2 domain of the LSU rRNA were located in segments that corresponded to the hairpins in the secondary structure (Sipiczki et al., 2013). Based on that observation only these segments were used here for structural analysis. In the case of ITS1–5.8S–ITS2, secondary structures were generated separately for ITS1 and ITS2. The procedures are described in the Supporting Information.

### 2.6 Mutagenesis, hybridization and examination of hybrids

Auxotrophic mutants were produced by nitrosoguanidine treatment and used for generating prototrophic hybrids by the replica‐plate method as described previously (Sipiczki et al., 2018). Briefly, line‐shaped cultures of mutants grown on YEA plates were replica‐plated onto fresh plates perpendicularly to each other to produce grids of prints. After 7 days of incubation, the grids were replica‐plated on SMA plates on which the mutants did not grow, but their hybrids formed at the intersections could grow. The hybrids were tested for sporulation, spore viability and mitotic segregation (described in the Supporting Information).

## 3 RESULTS

### 3.1 Analysis of database rDNA barcode sequences of strains related to *pulcherrima*‐clade type strains

**FIGURE 1:**
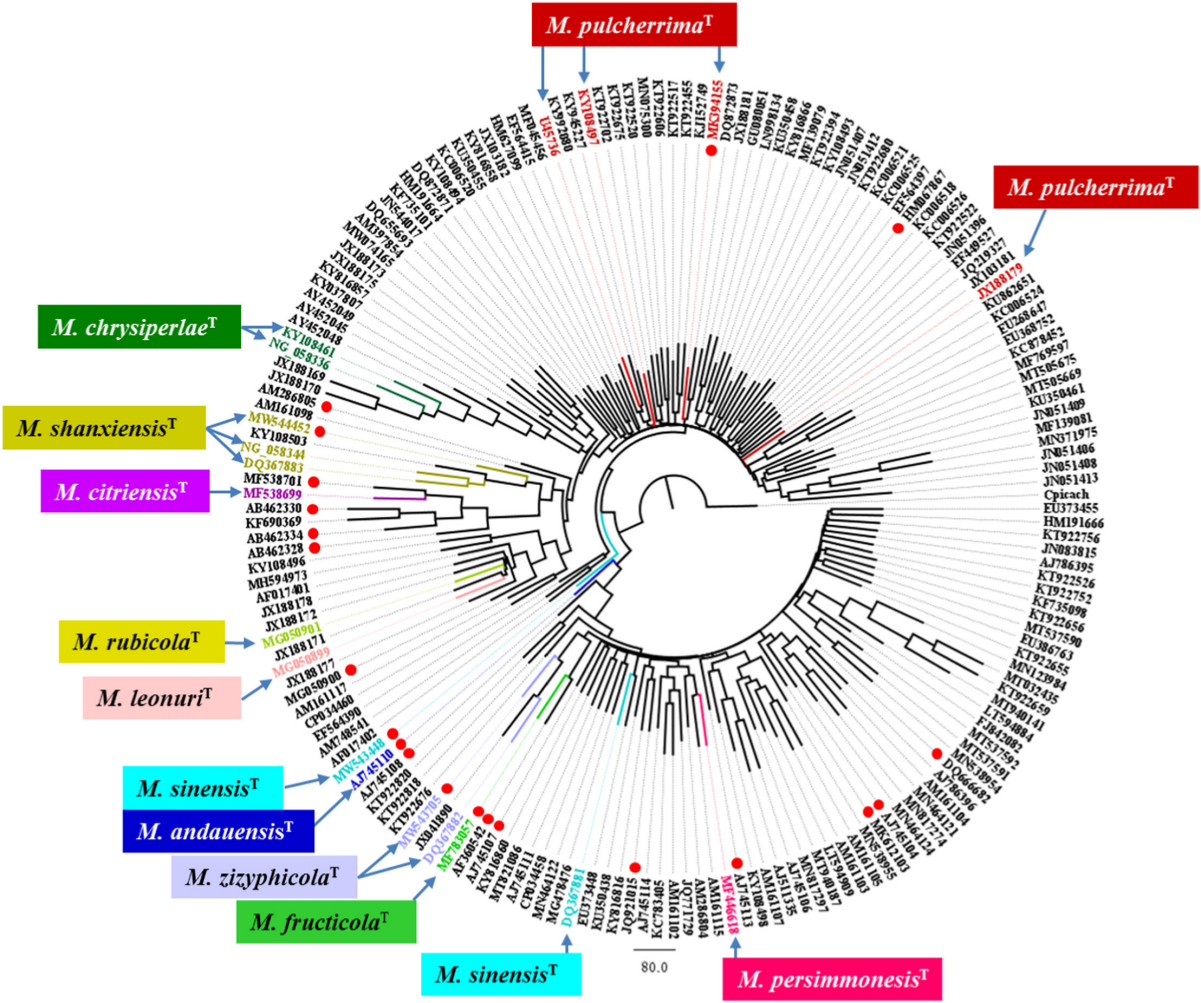
A tree derived from the neighbour‐joining analysis of database D1/D2 sequences. Type‐strain sequences are coloured. Red dots mark sequences containing ambiguous nucleotides. Outgroup: *Metschnikowia* (*Candida*) *picachoensis* CBS 9804^T^ (AY452039)

Over the past two decades, high numbers of D1/D2 and ITS1–5.8S–ITS2 sequences have been deposited in public databases, the similarity of which with the sequences of the type strains of the species of the *pulcherrima* clade indicates that the organisms from which they were amplified are associated with the clade. In this study the INSDC databases were searched for sequences similar to those deposited under the taxonomic name of one or the other species belonging to the clade. The blast similarity search with a 499‐nt‐long segment of the U45736 sequence corresponding to the D1/D2 LSU rRNA domain of the *Metschnikowia pulcherrima* type strain NRRL Y‐7111^T^ (CBS 5833^T^) identified 220 sequences with full species names and many taxonomically unassigned sequences with names *Metschnikowia* aff. or *Metschnikowia* sp. Among the 220 hits, the least similar sequence (MN782343.1) differed from the query sequence by 21.36% (E: 4e‐111). A similar search with a 354‐nt long stretch of the ITS segment (NR_164379.1) of the *M*. *pulcherrima* type strain resulted in 156 hits with the highest difference of 12.20% (E: 5e‐118) (MN371849.1). Remarkably, the most distant sequences were deposited under the species name *M*. *pulcherrima* in both searches, whereas sequences of strains of other species of the clade were less different. These distance values exceed several times the distances found between many ascomycetous species and certain related genera (e.g., Kurtzman & Robnett, 1998; Kurtzman and Robnett, 2003; Nilsson et al., 2008; Vu et al., 2016). However, the similarity values decreased continuously between the query sequences and the most distant sequences without forming separate clusters that might represent distinct taxonomic units. This continuity may be due to shortcomings of sequence‐based strain identification procedures widely used by nontaxonomists. The general practice in the taxonomic identification of a strain is a search of the INSDC databases with its D1/D2 or ITS sequences for identical/similar sequences and the assignment of the strain to the species whose database sequence is found to be most similar. The sequence is then deposited in one of the INSDC databases under this taxonomic name without an expert taxonomic verification (for reviews of problems with INSDC sequences, see Xu, 2016 and Lücking et al., 2020). Since small differences are usually ignored, the new entries will gradually expand the species beyond its biological boundaries. This practice can obscure the interspecies barcode gaps. To circumvent this problem in this study, new blast searches were individually performed with sequences of each type strain. The database sequences that differed from the type‐strain sequences by not more than 5% were then subjected to phylogenetic analyses. None of the species formed clearly separated clusters on any of the trees. Figure 1 (TreeBASE No. 28355) shows the NJ tree of the selected D1/D2 sequences. The ML trees had only slightly different topologies.

### 3.2 Sequence ambiguity in the rDNA of the *pulcherrima*‐clade type strains

On the tree shown in Figure 1, the *M*. *pulcherrima* type strain NRRL Y‐7111^T^ (CBS 5833^T^) is represented by four sequences from which only two (JX188179 and KY108497) are identical. At the time of the blast search, the non‐*pulcherrima* type strains had only single sequences in the databases, but the *M*. *andauensis* CBS 10809^T^ and *M*. *fructicola* CBS 8853^T^ sequences had numerous ambiguous nucleotides that call into question the correspondence between their locations on the tree and their real phylogenetic positions. The sequences of the recently described *M*. *leonuri* CBS 15341^T^ and *M*. *rubicola* CBS 15344^T^ have perfectly matching D1/D2 domains and are located next to each other on the tree as if they were conspecific. For the type strains of the rest of the species (*M*. *citriensis* CICC33213^T^, *M*. *persimmonesis* KIOMG15050^T^, *M*. *shanxiensis* CBS 10359^T^, *M*. *sinensis* CBS 10357^T^ and *M*. *zizyphycola* CBS 10358^T^) cloned sequences were deposited in the databases (Kang et al., 2017; Liu et al., 2018; Xue et al., 2006). To determine whether this group of strains could also have heterogeneous rDNA repeats, the D1/D2 domains of the *M*. *shanxiensis*, *M*. *sinensis* and *M*. *zizyphicola* ex‐type strains were amplified in this study and the amplicons were sequenced directly, without cloning. The sequences obtained in this way had seven (*M*. *shanxiensis*: MW544452), 10 (*M*. *sinensis*: MW543448) and 14 (*M*. *zizyphicola*: MW543705) ambiguous positions. The positions of these sequences on the tree shown in Figure 1 do not coincide with those of the clones.

The *M*. *pulcherrima* type strain also has slightly different ITS sequences (KY104205, JX188179 and JX188180) in the databases. Since no ITS sequences were available in the databases for the *M*. *andauensis* and *M*. *fructicola* type strains and because cloned sequences were provided for the rest of the type strains, I amplified the ITS regions of five ex‐type strains and sequenced the amplicons. Despite repeated attempts, the chromatograms of *M*. *andauensis* and *M*. *fructicola* were not readable in ITS1 and numerous positions in ITS2 were ambiguous. The sequences of the other strains had three (*M*. *zizyphicola*: MW543705) and four (*M*. *shanxiensis*: MW543708; *M*. *sinensis*: MW543446) variable positions.

### 3.3 Organization and sequence heterogeneity of rDNA in genome sequences

The publicly available *pulcherrima*‐clade genome sequences are diverse in genome size and in the quality of assembly (reviewed in Sipiczki, 2020), which hinders determination of the copy number and the exact location of rDNA repeats and other genes. The blast search for ITS and D1/D2 sequences performed in this study also revealed high repeat diversity (Table 2). The number of repeats in the genomes varied between four and 39 (including those containing truncated D1/D2 or ITS segments). The most fragmented genome sequence (*M*. aff. *pulcherrima* UCD127: 7,988 contigs) had the lowest number of repeats. None of the seven genomes had long continuous arrays. rDNA was present in six out of the 12 contigs of the *M*. *citriensis* type strain and in five out of the seven chromosomes of *M*. aff. *pulcherrima* APC 1.2. Most repeats were singletons or only occurred in pairs. All D1/D2 sequences of the *M*. *fructicola* type strain genome differed from the sequence deposited in the databases as the barcode on which the description of the species was based. The single complete ITS region of the *M*. *persimmonesis* genome sequence differed by 4.79% from the database type material sequence of the species and only by 2.54% from the *M*. *zizyphicola* type material sequence. None of the repeats of the *M*. *citriensis* genome were identical with the database type material sequences. The most divergent repeat differed from them by 7.87% (ITS) and 1.6% (D1/D2) but only by 0.85% from the ITS type material sequence of *M*. *shanxiensis* and 0.4% from the D1/D2 sequence of *M*. *rubicola*. These incongruities demonstrate how misleading the use of cloned sequences for species delimitation can be. Remarkably, high proportions of repeats (17%–75%) had incomplete ITS and/or D1/D2 segments.

**TABLE 2:** Organization of rDNA in assembled genome sequences

		Genome size^b^		Location of rDNA and the number of repeats^b^			
				ITS–5.8S–ITS2		D1/D2	
GenBank accession no.	Strain^a^	Length	Number of chromosomes/contigs	Complete	Truncate	Complete	Truncate
GCA_009746055.1	*M*. *citriensis* FLO1^T^= CICC33213^T^	25,739,826	12 contigs	Contig 2: 6 Contig 4: 6 Contig 5: 5 Contig 7: 17 Contig 10: 1 Contig 11: 1	Contig 1: 2 Contig 7: 1	Contig 2: 6 Contig 4: 6 Contig 5: 5 Contig 7: 17 Contig 10: 1 Contig 11: 1	Contig 1: 1 Contig 5: 1 Contig 7: 1 Contig 9: 1 Contig 10: 1
GCA_000317355.2	*M*. *fructicola* 277^T^=CBS 8853^T^	26,126,100	93 unitigs	Unitig 32: 1 Unitig 83: 1 Unitig 150: 1 Unitig 187: 2 Unitig 199: 2 Unitig 213: 1	Unitig 9: 1 Unitig 19: 1 Unitig 189: 1 Unitig 191: 2	Unitig 32: 1 Unitig 83: 1 Unitig 150: 1 Unitig 187: 2 Unitig 199: 2 Unitig 213: 1	Unitig 9: 1 Unitig 19: 1 Unitig 185: 1 Unitig 189: 1 Unitig 191: 1
GCA_003017285.1	*M*. aff. *fructicola* AP47	26,177,974	126 unitigs	Unitig 32: 1 Unitig 83: 1 Unitig 150: 1 Unitig 187: 2 Unitig 199: 2 Unitig 213: 1	Unitig 9: 1 Unitig 19:1 Unitig 189: 1 Unitig 191: 2	Unitig 32: 1 Unitig 83: 1 Unitig 150: 1 Unitig 187: 2 Unitig 199: 2 Unitig 213: 1	Unitig 9: 1 Unitig 19: 1 Unitig 185: 1 Unitig 189: 1 Unitig 191: 1
GCA_003123635.1	*M*. aff. *pulcherrima* UCD127	17,120,409	7988 contigs	NODE 3313: 1	NODE 1426: 1 NODE 2102: 1 NODE 2611: 1	NODE 3313: 1	NODE 2102: 1 NODE 2611: 1
GCA_004217705.1	*M*. aff *pulcherrima* APC 1.2.	15,801,215	7 chromosomes	Chr. I: 1 Chr. II: 2 Chr. III: 2 Chr. V: 2 Chr. VI: 1	Chr. I: 3 Chr. IV: 1	Chr. I: 1 Chr. II: 2 Chr. III: 2 Chr. V: 2 Chr. VI: 1	Chr. I: 3 Chr. IV: 1
GCA_014905795.1	*M*. *persimmonesis* KIOM G15050^T^	16,473,584	16 contigs	Contig 4: 1	Contig 3: 2 Contig 10: 1	Contig 1: 1 Contig 4: 1	Contig 2: 1 Contig 3: 2 Contig 10: 1

^a^ T, type strain.

^b^ It has to be taken into consideration that the genome sequences differ in the quality of assembly.

### 3.4 Cloned ITS and D1/D2 barcode segments show high intragenomic rDNA diversity

**FIGURE 2:**
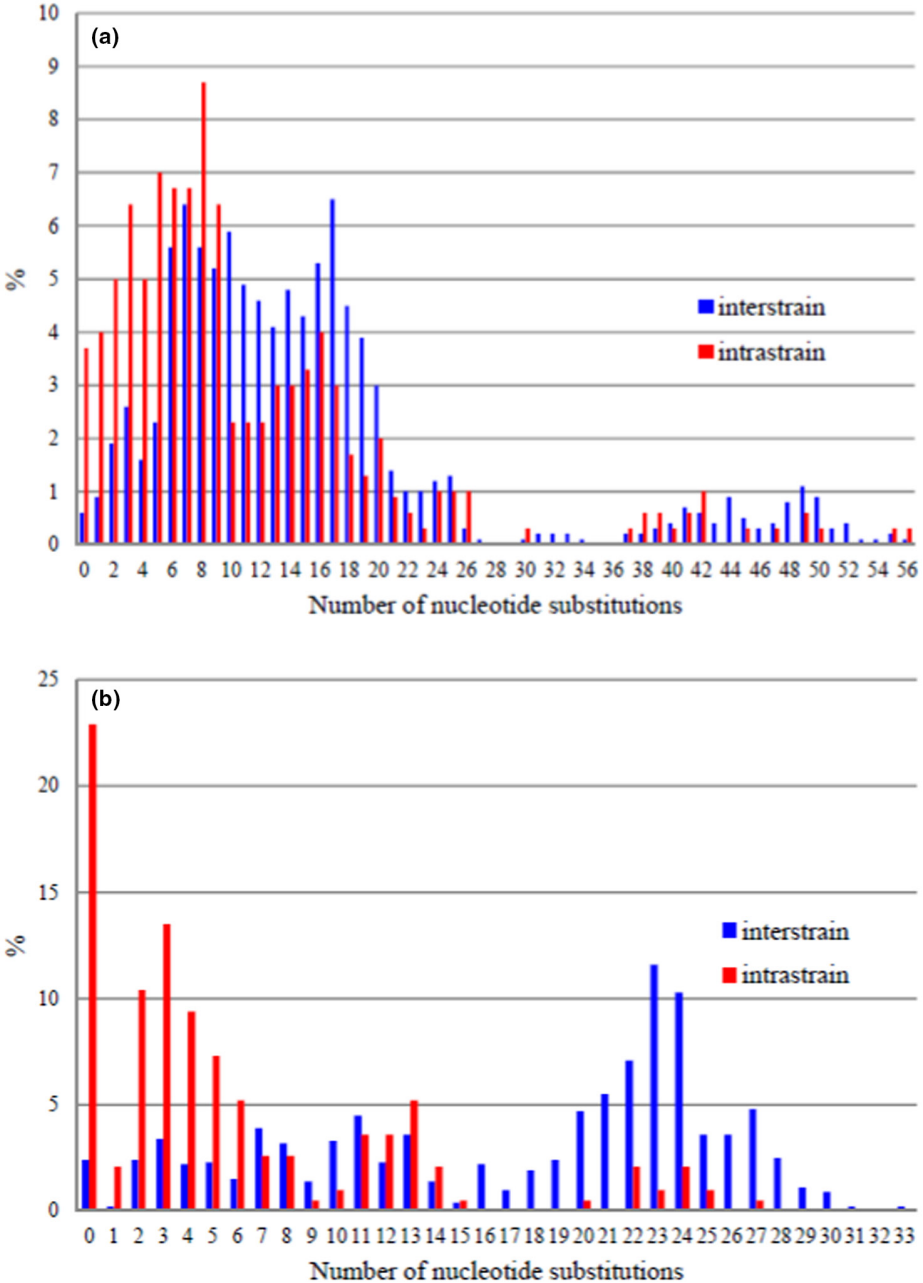
Distribution of pairwise distances among cloned rDNA barcode sequences. (a) D1/D2 domain sequences. (b) ITS1–5.8S–ITS2 sequences

The low number of rDNA repeats found in the genome sequences indicates that large parts of the rDNA were not assembled and thus these assemblies do not show the entire intragenomic diversity. To gain more detailed information on the intragenomic repeat diversity, individual ITS and D1/D2 segments were cloned and sequenced in this study from ex‐type cultures of six species (Table S1). All strains were shown to harbour ITS and D1/D2 sequences not seen in the whole‐genome sequences and the overall diversity among the clones was higher than among the genomic repeat sequences. The maximum intragenomic differences ranged between 7 and 27 nt (ITS, 320 nt) and between 18 and 56 nt (D1/D2, 499 nt) (Figure 2).

### 3.5 Nonrandom distribution of variable positions in the rDNA barcode sequences

Previous studies on a limited number of cloned sequences revealed that the intragenomic rDNA diversity of the *M*. *andauensis* and *M*. *fructicola* type strains was due to nucleotide substitutions at particular positions (Sipiczki et al., 2013, 2018). In this work, with the aim to examine the distribution of the variable positions in more strains, 92 ITS sequences and 118 D1/D2 sequences cloned from ex‐type cultures or found in whole‐genome sequences were aligned.

**FIGURE 3:**
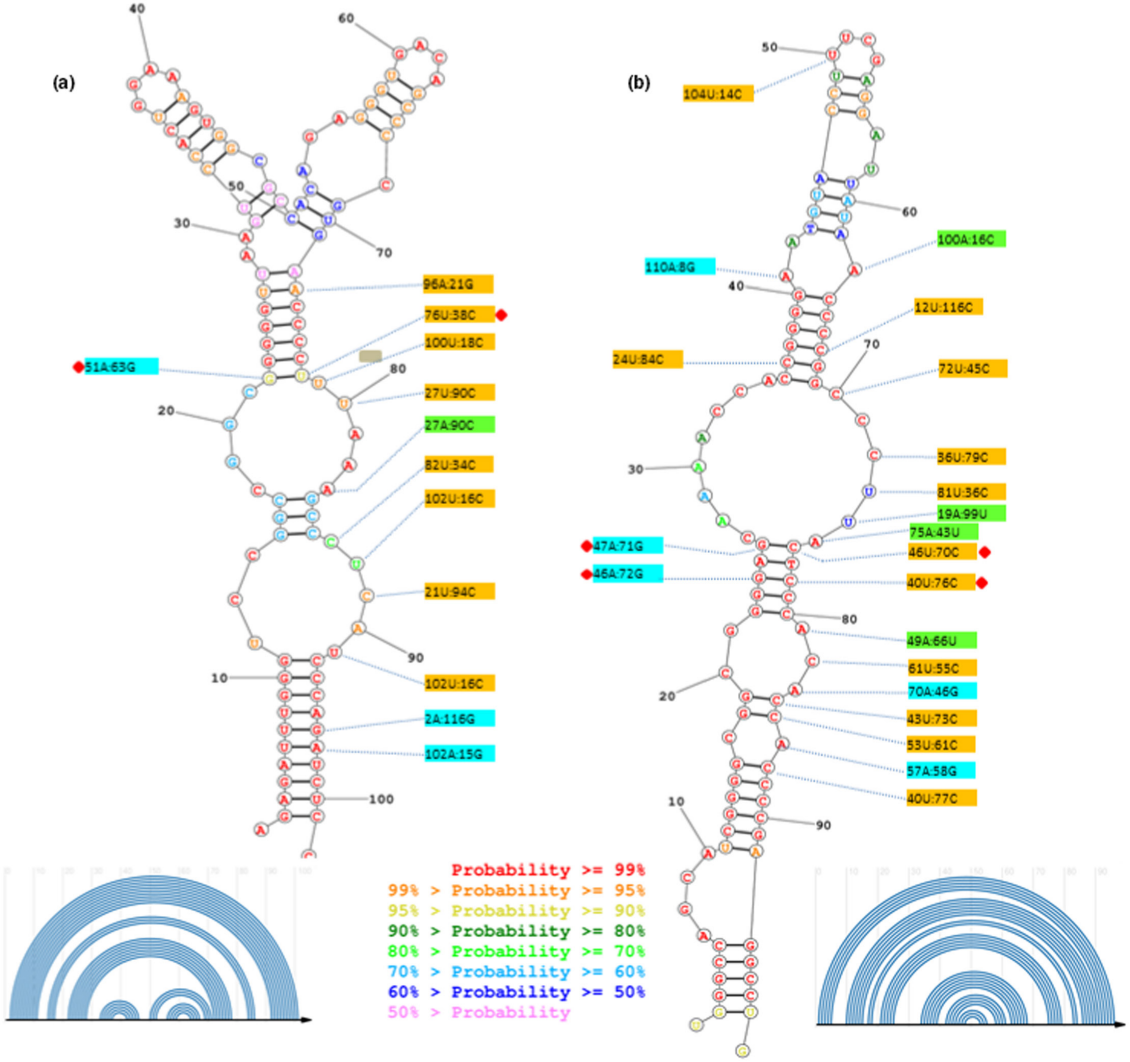
Predicted secondary structures of D1 and D2 hairpin–stem loops of the transcript of U45736 of the *Metschnikowia pulcherrima* type strain. (a) D1 loop. (b) D2 loop. In the lower parts of both panels, an R‐chie diagram shows the positions of the pairing segments. Variable (dimorphic) sites are marked with flags. For each site, the proportion of nucleotides in all sequences cloned from the type strains or found in the genome sequences is shown in the corresponding flag. Yellow: pirimidin transition; blue: purin transition; green: transversion. Sites of concerted compensatory base changes (CBCs) are marked with red symbols

In the D1/D2 alignments, nucleotide variability was detected in segments which corresponded to the D1 and D2 hairpin loops (stem loops) of the secondary structures generated from transcribed sequences (Figure 3). Only sites are marked in the figure at which more than two sequences differed from the rest. Within the loops, the variable sites grouped mainly in the nonpaired segments of the back‐folding strands of the loop stems where their changes probably do not affect significantly the folding of the RNA molecules. Substitutions in the paired segments were less abundant and could be divided into two types: substitutions only in the back‐fold stretch or in both stretches at pairing positions. In the former case changes mainly occurred whose effect on the secondary structure can be neutralized by wobble pairing. In RNA helices, guanine can pair not only with cytosine but also with uracil (Varani & McClain, 2000). Thus, alterations between C and U are structurally neutral if the pairing partner is G. Likewise, A–G transitions also can be tolerated if the matching partner is U. Pyrimidine transitions predominated, and transversion was rare. One pair of sites in the D1 loop and two pairs in the D2 loop appear to undergo concerted compensatory base changes (CBCs; marked with a red square in Figure 3). In CBCs, substitutions on one side of a pair are compensated for by substitutions on the other side to keep the nucleotides paired. However, certain sequences had mismatching base pairs in these positions. For example, 20 sequences had the mismatching A–C combination in positions 22 and 78 in the D1 loop. Since CBCs are thought to be essential for the correct structure and activity of the RNA (e.g., Rousset et al., 1991; Wolf et al., 2013), these sequences may be inactive pseudogenes. Substitutions outside of the loops were rare, usually occurred in single sequences and might be attributed to amplification errors (e.g., Taq error). The only significant exception (26 U:91A) was a variable site located behind the D2 loop.

**FIGURE 4:**
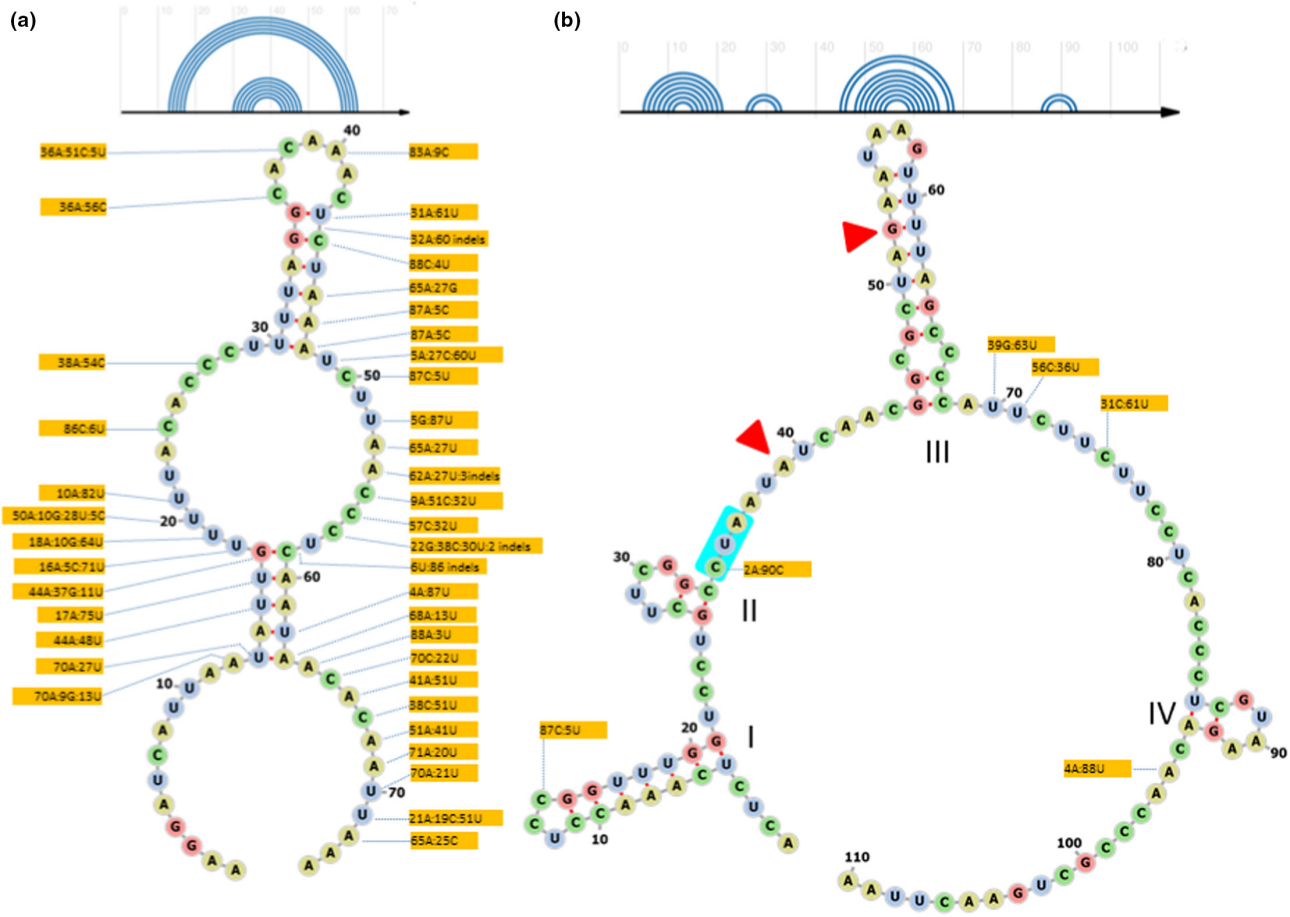
Predicted secondary structures of the ITS1 and ITS2 segments of the transcript of NR_164379 of the *Metschnikowia pulcherrima* type strain. (a) ITS1 (minimum free energy: −2.50). (b) ITS2 (energy: −20.2). In the upper parts of both panels, an R‐chie diagram shows the locations of the pairing segments. Variable (di‐ and polymorphic) sites are marked with flags. For each site, the proportion of nucleotides in all sequences cloned from the type strains or found in the genome sequences is shown in the corresponding flag. The sum is <92 at positions where single‐nucleotide deletion were present in certain sequences. The conserved loops of ITS2 are labelled with Roman numerals. Red triangles mark the segments that correspond to the cut sites in *Saccharomyces cerevisiae* (Coleman, 2015). The segment that corresponds to the binding site of the Nop15 protein involved in the processing of ITS2 in *S*. *cerevisiae* (Granneman et al., 2011) is marked with a blue background

In the ITS1–5.8S–ITS2 alignments, ITS1 was much more variable than ITS2: 54% of the positions of ITS1 but only 5% of the positions in ITS2 showed variability. This difference could suggest that ITS1 is less subject to functional constraints and changes (evolves) at a higher rate. As in the case of the D1/D2 domains, positions located in the nonpairing areas appeared more prone to change than positions in the paired segments (Figure 4). However, unlike the D1 and D2 loops, in which the variable positions alternate between two nucleotides (single nucleotide dimorphism, SND sites), here both transitions and transversions were detected at numerous positions.

### 3.6 The rDNA barcode sequences form a continuous pool undivided by barcode gaps

**FIGURE 5:**
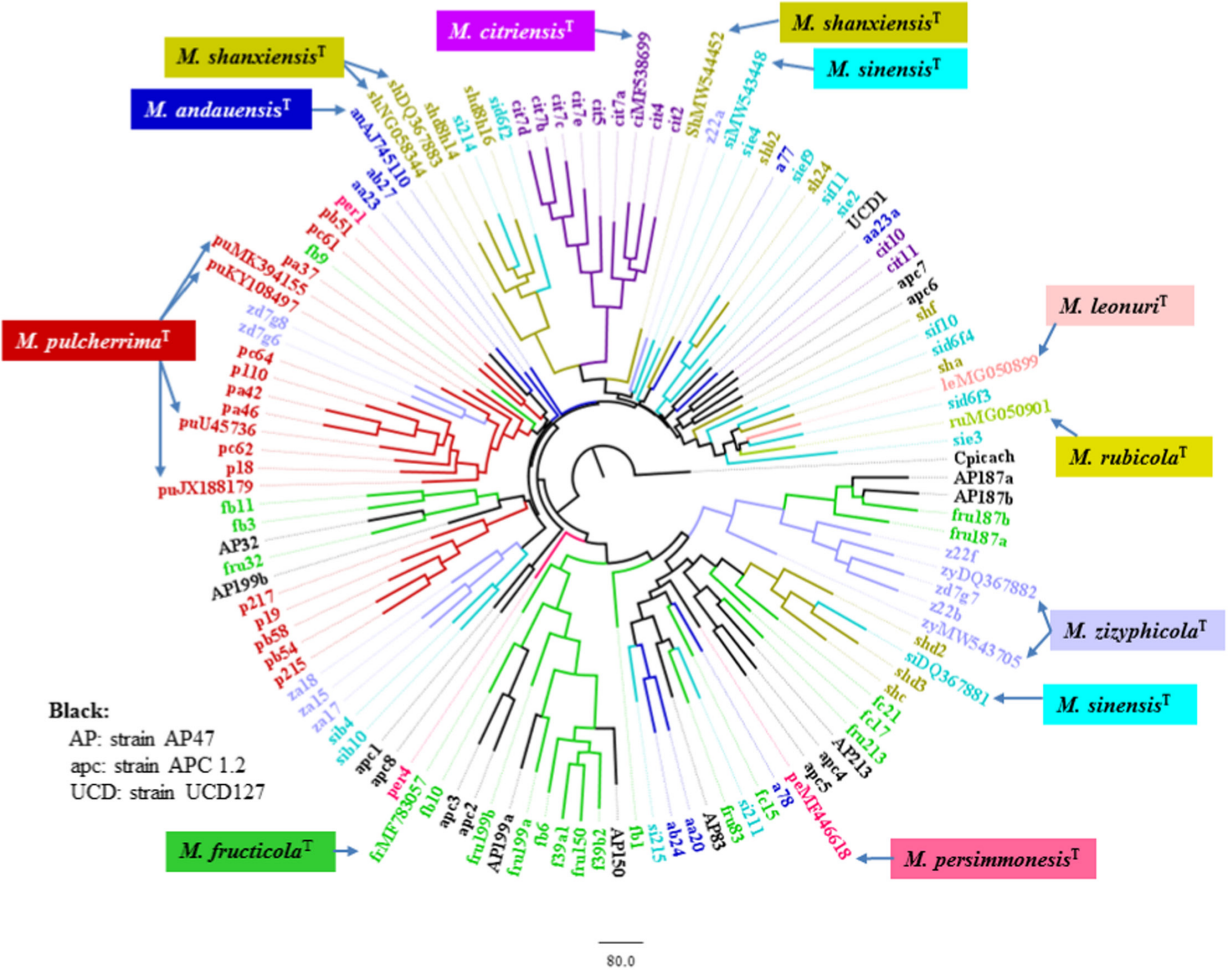
A tree derived from the neighbour‐joining analysis of the D1/D2 sequences. The flagged sequences are “taxonomic” sequences on which the delimitation of the species was based. The rest are cloned from the type strains or found in the genome sequences. Outgroup: *Metschnikowia* (*Candida*) *picachoensis* CBS 9804^T^ (AY452039). Each type strain is shown in a different colour. Black: genomes of nontype strains

To elucidate the evolutionary history of the repeats, the cloned and the genomic sequences as well as the database type‐material sequences were aligned and the alignments were analysed with NJ and ML methods. The topologies of the NJ and ML trees were congruent but the statistical support of the majority of the branches was very low. The sequences of the species did not form clearly distinct and compact clusters on either the D1/D2 (Figure 5) or the ITS (Figure S1) tree (TreeBASE Nos. 28358 and 28359).

### 3.7 Visualization of reticulation by phylogenetic network analysis

**FIGURE 6:**
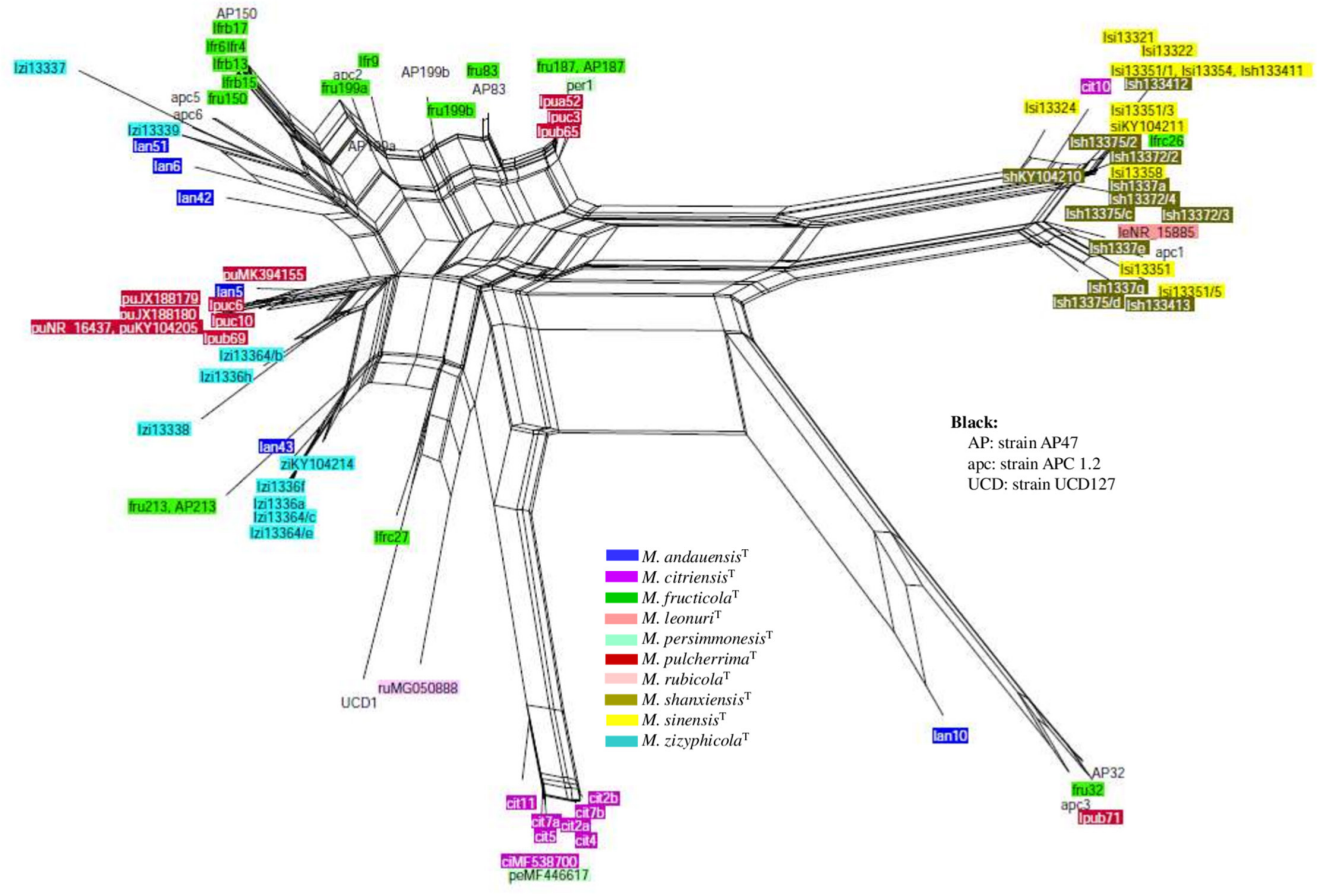
Neighbour‐net splits graph of all cloned and genomic ITS sequences. The scale bar represents the split support for the edges

**FIGURE 7:**
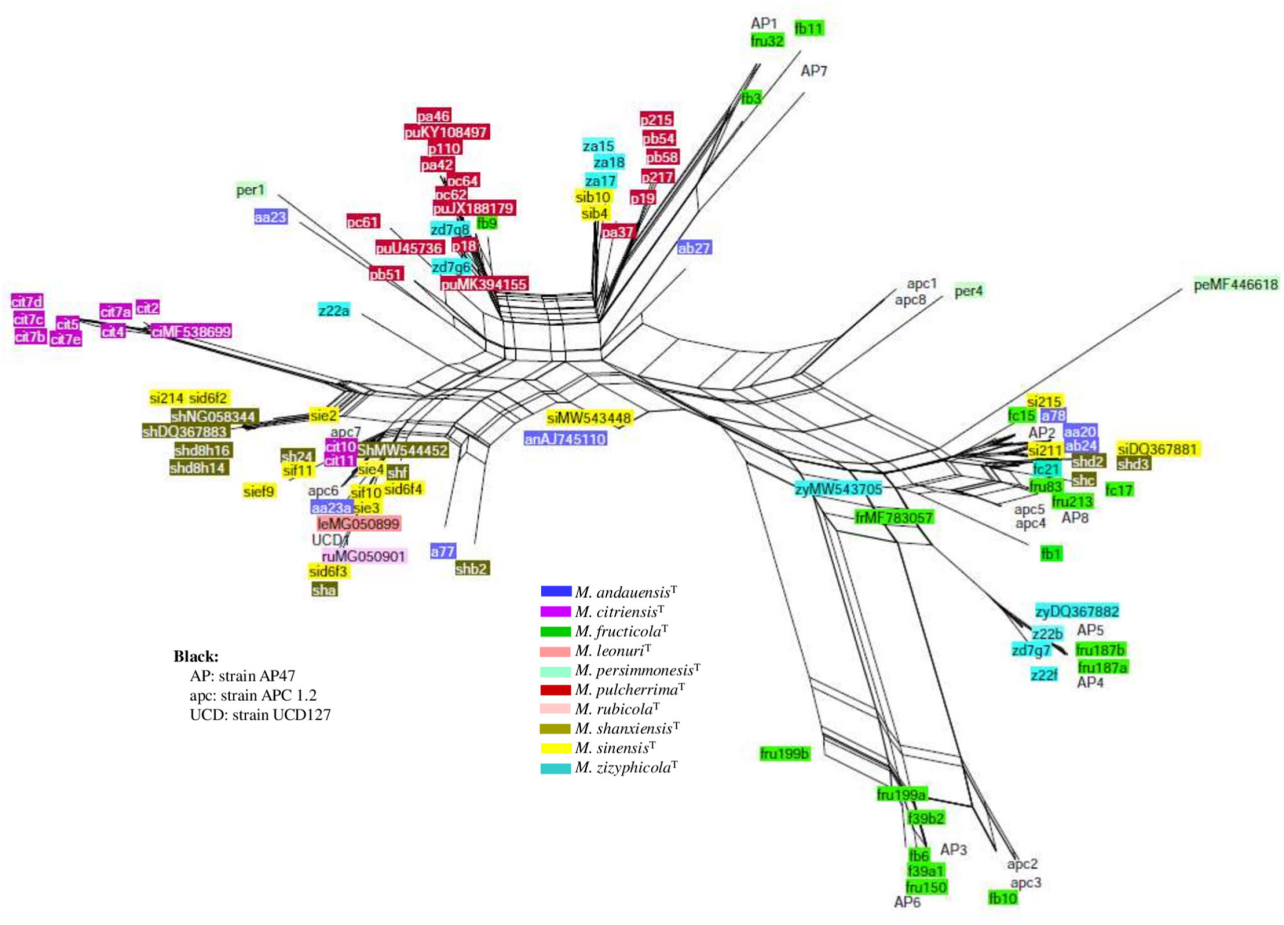
Neighbour‐net splits graph of all cloned and genomic D1/D2 sequences. The scale bar represents the split support for the edges

The intermixing of the sequences on the phylogenetic trees and the low statistical support of the branches indicated that the rDNA repeats of the strains did not evolve in a purely tree‐like manner. Interstrain interactions (reticulation) may also have been involved. Reticulation can be visualized by generating phylogenetic networks from the sequences. Therefore, from the sequence alignments neighbour‐net splits graphs were also generated. In a network, a pair of nodes can be linked by a single edge (tree‐like part) or a set of parallel edges depicting alternative evolutionary possibilities (reticulate part). The neighbour‐net splits graphs of the ITS (Figure 6) and D1/D2 sequences (Figure 7) were distinctly nontree‐like. Thus, reticulation was involved in the evolution of the rDNA repeats of the examined *pulcherrima*‐clade strains.

In the ITS network, the majority of the *M*. *shanxiensis* and *M*. *sinensis* sequences group together and are well separated from the majority of the *M*. *citriensis* sequences. In the D1/D2 network, they form a less compact cluster that is close to the major group of the *M*. *citriensis* sequences. It is noteworthy that although the *M*. *citriensis* sequences form a fairly compact group in both splits graphs, its genome has sequences that group with *M*. *shanxiensis* and *M sinensis* sequences, and the *M*. *persimmonesis* sequence MF446617 is among the *M*. *citriensis* sequences in the ITS network. The sequences of the type strains were highly intermixed in the parsimony networks as well (Figures S2 and S3).

### 3.8 Secondary barcode sequences of the type strains

*ACT1*, *RPB2*, *TEF1* and *EF2* barcode sequences have been deposited in the INSDC databases for eight type strains of the *pulcherrima* clade (Table 3). Most of these sequences have ambiguous nucleotides (marked with stars in the table), indicating that the strains have the relevant genes in more than one copies.

**TABLE 3:** Secondary barcode sequences (barcode segments of protein‐coding nuclear genes)

		Genes^b^			
Species	Strain^a^	*ACT1*	*RPB2*	*TEF1*	*EF2*
*M*. *andauensis*	CBS 10809^T^	AJ745122* KC859880*	KC859678*	MG050911* Clone MG595725^1^	MG655231*^1^ Clone MG655232^1^ Clone MG655233^1^ Clone MG655234^1^ Clone MG655235^1^ Clone MG655236^1^ Clone MG655237^1^
*M*. *fructicola*	CBS 8853^T^	AJ745127* KC859895	KC859693* Clone MW799687^1^ Clone MW799688^1^ Clone MW799689^1^ Clone MW799690^1^ Clone MW799691^1^ Clone MW799692^1^ Clone MW799693^1^ Clone MW799694^1^ Clone MW799695^1^	MG050908* Clone MG595726^1^	KC859816 7 clones of identical sequence^1^
*M*. *leonuri*	CBS 15341^T^		MG050965*	MG050914*	
*M*. *pulcherrima*	CBS 5833^T^	AJ745126* KC859911	KC859707*	MG050910*	AY497653 (CBS 610)
*M*. *rubicola*	CBS 15344^T^		MG050966	MG050916*	
*M*. *shanxiensis*	CBS 10359^T^	KC859914* MW847421^1^	KC859710* MG050972*	MG050912*	KC859833*
*M*. *sinensis*	CBS 10357^T^	KC859917* MW847420^1^	KC859713*		KC859836*
*M*. *zizyphicola*	CBS 10358^T^	KC859920* MW847422^1^	KC859716*	MG050909*	KC859838*

^a^ T, type strain.

^b^ *There are ambiguous nucleotides in the sequence; ^1^sequenced in this study.

#### 3.8.1 *ACT1* (actin)

The two sequences of the *M*. *andauensis* type strain have eight ambiguous nucleotides in matching positions, which implies that this strain has two or more different *ACT1* genes. Two sequences are available for each of the *M*. *pulcherrima* and *M*. *fructicola* type strains and one of them has ambiguous positions in both strains. The single *M*. *shanxiensis*, *M*. *sinensis* and *M*. *zizyphicola* sequences have one or two such nucleotides. To determine if their presence was due to sequencing errors, the relevant segments of their *ACT1* genes were resequenced. Only the *M*. *zizyphicola* sequences had an ambiguous nucleotide. When compared with each other, the *M*. *fructicola* and *M*. *pulcherrima* sequences were identical and differed by one substitution from the *M*. *shanxiensis* and *M*. *sinensis* sequences, which also were identical. The *M*. *zizyphicola* sequence differed from the former pair by two and from the latter pair by one nucleotide.

#### 3.8.2 *EF2* (elongation factor 2)

Prior to the beginning of this study, partial *EF2* sequences had been deposited in the databases for four type strains of the clade. The *M*. *shanxiensis*, *M*. *sinensis* and *M*. *zizyphicila* sequences contained two to three ambiguous nucleotides but the *M*. *fructicola* sequence was clean. In this study, the *EF2* barcode segments of the ex‐type strains of these species and *M*. *andauensis* were sequenced. The new sequences were identical to those available in the databases and the *M*. *andauensis* sequences had four variable positions, two of which corresponded to the variable positions of the *M*. *shanxiensis* and *M*. *sinensis* sequences. Thus, four out of the five strains seem to have two or multiple *EF2* genes that differ in sequence. To test this assumption, individual fragments were cloned from amplicons amplified from the *M*. *andauensis* and *M*. *fructicola* strains (Table 3). While the seven *M*. *fructicola* clones had identical sequences, the six *M*. *andauensis* clones formed two groups differing at the sites where the database sequence had ambiguous nucleotides. One group was identical to the *M*. *fructicola* sequence. The other group was more similar to the pair *M*. *shanxiensis* – *M*. *sinensis*. These results indicate that the *M*. *andauensis* type strain has two *EF2* genes whose phylogenetic roots (histories) are different.

#### 3.8.3 *RPB2* (RNA polymerase II second largest subunit)

All but one barcode sequences of the eight type strains shown in Table 3 have high numbers of ambiguous nucleotides. The exception is that of *M*. *rubicola*. To determine whether the ambiguity was due to sequencing errors or to intragenomic diversity, fragments were cloned from the amplified segment of the *M*. *fructicola RPB2* gene. The 11 cloned sequences were clean and could be divided into three groups differing at 19 positions. So the *M*. *fructicola* type strain has at least three different *RPB2* gene sequences. Assuming that the ambiguities of the *RPB2* sequences of the rest of the strains were also attributable to intragenomic diversity, it can be concluded that all but one type strain of the clade have multiple *RPB2* genes.

#### 3.8.4 *TEF1* (Translational elongation factor EF‐1 alpha)

The most diverse barcode gene is *TEF1*. The sequences listed in Table 3 have many (up to 30) ambiguous nucleotides, most of them being at matching sites in several sequences. The table also contains *M*. *andauensis* and *M*. *fructicola* sequences cloned in this work. Neither clone has ambiguous nucleotides, so the ambiguities in the database sequences were unlikely to be due to sequencing errors.

### 3.9 Genes of the secondary barcodes in genome sequences

Using the barcode sequences as queries in a blast search in the genome sequence of the *M*. *fructicola* type strain, I found two *ACT1*, one *EF2*, one *RPB2* and six *TEF1* genes (Table 4). The *ACT1* genes were identical in sequence and contained segments which were identical to the database barcode sequence KC859895. The *EF2* sequence was also identical to the database sequence. In contrast, the *RPB2* gene differed both from the database sequence and from all cloned sequences. The presence of a single copy in the genome sequence and its difference from the cloned sequences might be due the incompleteness of the genome sequence or to assembly errors. The *TEF1* genes formed four groups which differed from each other by up to 22 substitutions. As the barcode database sequence contained numerous ambiguous positions, all *TEF1* sequences of the genome differed from it. One of the genomic sequences had a segment which was identical with the cloned barcode sequence MG595726.

**TABLE 4:** Number of the selected genes in genome sequences

Gene	Type strains, genome sequence accession number (genome size)			Non‐type strains, genome sequence accession number (genome size)		
	*M*. *citriensis* GCA_009746055.1 (25,739,826)	*M*. *fructicola* GCA_000317355.2 (26,126,100)	*M*. *persimmonesis* GCA_014905795.1 (16,473,584)	APC1.2 GCA_004217705.1 (15,801,215)	AP47 GCA_003017285.1 (26,177,974)	UCD127 GCA_003123635.1 (17,120,409)
Barcode						
*ACT1*	1	2	1	1	2	1
*EF2*	1	1	1	1	1	1
*RPB1*	1	2	1	1	2	2
*RPB2*	2	1	1	1	1	1
*TEF1*	3	6	1	2	6	2
Pulcherrimin synthesis						
*PUL1*	2	2	1	1	3*	3*
*PUL2*	2	2	1	1	2	1
*PUL3*	2	2	1	1	2	2*
*PUL4*	2	2	1	1	2	1
*SNF2*	2	1	1	1	1	1*
*STH1*	2	2	1	1	2	2*
Chitinase						
*CTS1‐A*	2	2	1	1	2	2
*CTS1‐B*	2	2	1	1	2	2*
*CTS1‐C*	2	2	2	1	2	2*
*CTS2*	1	2	2	1	2	2*
Exoglucanase						
*KRE6‐1*	1	2	1	1	2	2*
*KRE6‐2*	1	2	1	1	2	2*
*UTR2‐1*	1	2	1	1	2	?
*UTR2‐2*	1	2	1	1	2	2*
*SCW‐1*	2	1	1	1	1	1
*SCW‐2*	1	1	1	1	1	3*
*SCW‐3*	2	2	1	1	2	2
*SCW‐4*	2	2	1	1	2	2*
Endoglucanase						
*ENG1‐1*	1	1	1	1	1	1
*ENG1‐2*	1	1	1	1	1	1
*ENG1‐3*	2	2	2	1	2	4*

* gene fragments (truncate genes).

The genomes of the type strains of *M*. *citriensis* and *M*. *persimmonesis* as well as four strains (AP47, APC 1.2, Bath1 and UCD 127) of uncertain taxonomic position within the *pulcherrima* clade were also sequenced and the sequences are publicly available. The search of these sequences identified variable copy numbers of the genes (Table 4). The larger genomes had higher numbers of certain genes. However, when comparing the copy numbers of genes, it should be taken into account that in the highly fragmented UCD 127 genome sequence, the same gene can be identified more than once as fragments located on different contigs. Interestingly, in genomes having multiple gene copies, the intragenomic diversity of certain genes was higher than the interstrain (interspecies) diversity. For example, in the case of *RPB2*, the difference between the *M*. *citriensis* genes (127 substitutions) was higher than the difference of one of them (77 substitutions) from the *M*. *fructicola* gene. Comparison of the six *TEF1* genes revealed high intragenomic diversity and a closer relationship of certain *M*. *fructicola* genes and their counterparts in *M*. *citriensis* and *M*. *persimmonesis*. For example, the gene located on unitig 50 is somewhat more similar to one of the *M*. *citriensis* genes (four substitutions) than to the least different *TEF1* gene (five substitutions) within the *M*. *fructicola* genome. On the NJ tree shown in Figure S4 (TreeBASE No. 28405) the *TEF1* genes of the three type strains are located intermixed.

### 3.10 Admixed (chimeric) genomes

The high intragenome diversity of the barcode sequences and the lack of clear barcode gaps between the ex‐type strains indicate that their genomes consist of mosaics of diverse origins. To further investigate the genome structures, the analysis was extended to a larger set of genes. In addition to the barcoding genes, 30 genes supposedly involved in the antimicrobial antagonism of the strains (Sipiczki, 2020) or their sexual interactions and 100 randomly selected genes were chosen for the investigation. The genes of strain APC 1.2 were used as query sequences because this strain had the smallest genome, was assembled to the chromosome level and had single copies of almost all genes. Searching the other genomes with the coding regions of its genes for similar sequences identified all genes in all genomes (Table 4). The larger genomes had more than one copy of most genes, which indicates that the higher genome size might be (at least partially) due to segmental diploidy, and in the case of *TEF1* even segmental polyploidy. To determine whether they were paralogues generated by duplication or orthologues (homeologues) of different origin, their entire coding regions were downloaded and compared with each other. Table 5 shows the result of the comparison of 35 genes of the *M*. *citriensis*, *M*. *fructicola* and *M*. *persimmonesis* genomes with their counterparts in the APC 1.2. genome as outgroups. No clear tendency could be seen in the relationships of the three type strains. When a strain had more than one copy of a gene, the copies always had different sequences and were frequently less similar to each other than to their counterparts in other strains. Similar results were obtained from the analysis of the 100 randomly selected genes (data not shown). The higher intergenome (interstrain) similarity corroborates the hypothesis that the genomes are composed of mosaics of different origins. The genes involved in pulcherrimin production (the *PUL* cluster and the regulators *SNF2* and *STH1*) could be divided into two groups in the *M*. *citriensis* genome. One group was strikingly different from all their counterparts in the other genome sequences. This group might have been acquired from a phylogenetically divergent strain of the clade.

**TABLE 5:** Similarity of genomic sequences to APC 1.2 genes

Gene	Percentage similarity to the APC 1.2 gene					
	Most similar		Less similar		Least similar	
*ACT1*	99.63		99.54		99.45	
					99.45	
*EF2*	99.28		99.12		98.03	
*RPB1*	98.58		98.42		97.80	
			97.82			
*RPB2*	98.30		98.11		96.60	
			97.30			
*TEF1*						
(Chr.V)	99.93		99.54		97.46	
			99.34			
			99.47			
			99.27			
			98.96			
			97.68			
			97.54			
(Chr. VI)	99.71		99.64		97.32	
	99.71		97.54		97.32	
			97.53			
			97.39			
*CTS1‐A*	97.07		95.38		91.49	
			91.55			
*CTS1‐B*	97.49		97.22		96.47	
			96.55			
*CTS1‐C*	96.60		96.52		96.08	
			96.44			
*CTS2*	97.06		96.69		95.78	
			96.05		95.78	
*KRE6‐1*	98.06		98.01		95.40	
			96.93			
*KRE6‐2*	97.98		97.22		95.81	
			96.52			
*UTR1‐1*	98.75		98.45		98.23	
			98.38			
*UTR1‐2*	97.73		97.66		96.64	
			96.93			
*SCW‐1*	99.90		99.62		98.10	
			99.52			
*SCW‐2*	97.56		96.11		95.79	
*SCW‐3*		94.61		94.40		90.36
				93.23		
				92.63		
*SCW‐4*		97.30		97.23		91.95
				97.09		
				97.03		
*ENG1‐1*		98.64		97.31		95.48
*ENG1‐2*		98.23		98.09		97.59
*ENG1‐3*		98.04		97.82		86.88
				96.69		
				96.62		
				9056		
*ENG2*		97.57		97.05		96.77
*PUL1*		98.33		97.89		95.27
				97.81		
				97.60		
*PUL2*		98.26		97.85		94.86
				97.69		
				97.64		
*PUL3*		97.81		97.70		95.08
				97.49		
				97.49		
*PUL4*		98.35		98.02		95.19
				97.87		
				97.44		
*SNF2*		99.20		98.50		95.75
				98.35		
*STH1*		98.90		98.01		95.05
				97.95		
				97.95		
*MATA1*		100		99.72		
		100				
*MATALPHA1*		100		98.02		
*MATALPHA2*		98.83		98.09		96.77
				97.94		
				97.65		
*MF(ALPHA) L*		98.62		98.23		92.15
				96.91		92.15
				95.81		
*MF(ALPHA) S*		94.51		94.01		93.06
*STE2* Alpha‐factor receptor		97.81		97.72		95.72
				97.09		
*STE3* A‐factor receptor		98.55		98.37		97.92
				98.10		

Note

Blue: *M*. *fructicola* ^T^; green: *M*. *citriensis* ^T^; yellow: *M*. *persimmonesis* ^T^.

### 3.11 Sexual compatibility of strains: Hybridization and hybrid segregation

**FIGURE 8:**
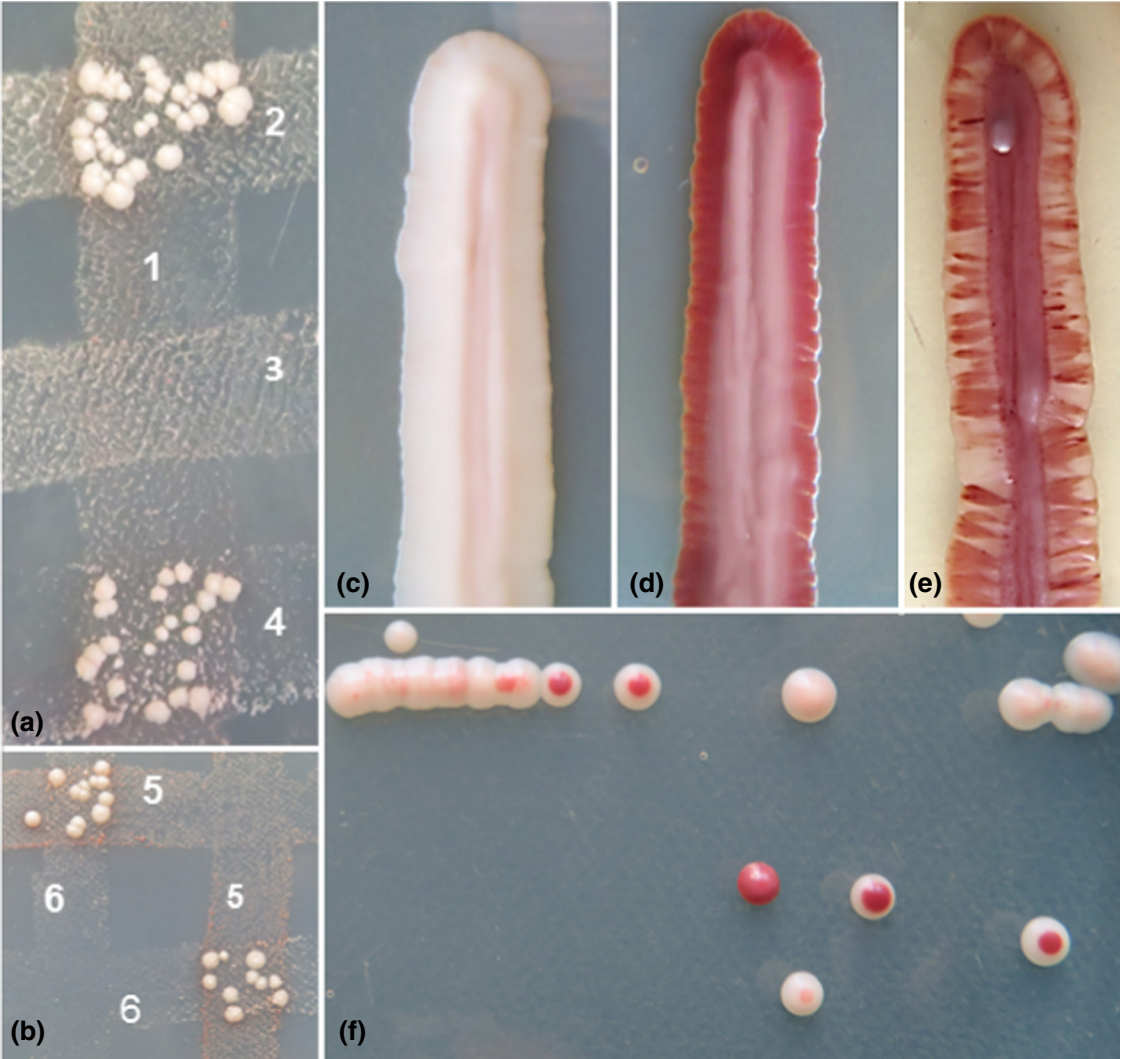
Hybridization and segregation. Growth of prototrophic hybrid colonies at the intersections of (a) the auxotrophic *Metschnikowia shanxiensis* mutant with auxotrophic *Metschnikowia pulcherrima* mutants and (b) two auxotrophic *Metschnikowia andauensis* mutants on minimal medium SMA. Colour of hybridizing strains (c) 1605 *M*. *shanxiensis*, (d) 1593 *M*. *pulcherrima* lys^−^ and (e) their segregating hybrid on the complete medium YEA supplemented with 0.02 mg FeCl_3_. (f) Segregant colonies formed by cells of the hybrid culture on the latter mediu *M*. Mutant strains: (1) 1605 *M*. *shanxiensis* asp^−^, (2) 1591 *M*. *pulcherrima* his^−^, (3) 1592 *M*. *pulcherrima* ade^−^, (4) 1593 *M*. *pulcherrima* lys^−^, (5) 1598 *M*. *andauensis* arg^−^ and (6) 1599 *M*. *andauensis* try^−^

The segmental mosaicism of the genomes and the reticulation of the rDNA repeats imply that exchange of genetic information might have played a role in the evolution of the type strains of the clade. To examine the sexual compatibility of the ex‐type strains, auxotrophic mutants were generated in this study and tested for their ability to form prototrophic hybrids with *M*. *pulcherrima* auxotrophic strains (Figure 8). Table 6 shows that the five type strains involved in the tests formed viable hybrids with the *M*. *pulcherrima* mutants of *alpha* mating type. However, many mutants did not hybridize with any of the *M*. *pulcherrima* auxotrophic mutants (not shown in Table 6). This might be due to the low mating potential of the *M*. *pulcherrima* tester of mating type *a* (it hybridized very poorly even with conspecific *alpha* mutants) or to mating deficiency in the partners. When the mutants of the non‐*pulcherrima* type strain were tested for hybridization with each other, evidence was found for both possibilities. For example, the *M*. *andauensis* mutant showing a poor hybridisation activity with the *M*. *pulcherrima* mutant of *a* mating type, hybridized very efficiently with the other *M*. *andauensis* mutant (Table 6), but the mutants of *M*. *fructicola* did not form hybrids with each other. Nevertheless, the viability of the hybrids of the type strains with the *M*. *pulcherrima* type strain implies that the species of the clade are not isolated by prezygotic reproductive barriers. To determine if they are isolated postzygotically, selected hybrids were tested for sporulation and spore viability. Except for the combination *M*. *pulcherrima* × *M*. *andauensis* (3%–16% asci), the hybrids sporulated very poorly (<1%) and no spore germination was observed on fresh complete medium. As ascospores are the yeast equivalents of gametes of plants and animals, these results hint at postzygotic sterility barriers (failure to form viable gametes) between the tested strains. If no viable gametes are produced, no meiotic segregation can take place and the hybrid genomes could remain stable. However, when the hybrids of mutants differing in the intensity of pigment production were inoculated on a medium supplemented with FeCl_3_, sectored colonies were formed (Figure 8). Spreading samples of the hybrid cultures onto plates of this medium resulted in mixtures of differently pigmented colonies. Thus, the hybrids can segregate during the vegetative propagation of cells (mitotic segregation), which can lead to the formation of chimeric genomes composed of mosaics of parental genomes.

**TABLE 6:** Hybridization of auxotrophic mutants

Strain^a^	Marker	*M*. *pulcherrima* ^T^		
		11–1591 α his^−^	11–1592 a ade^−^	11–1593 α lys^−^
*M*. *pulcherrima* ^T^				
11‐1591	α his^−^	−	+	−
11‐1592	a ade^−^	+	−	+
11‐1593	α try^−^	−	+	−
*M*. *andauensis* ^T^				
11‐1598	arg^−^	−	(+)	−
11‐1599	try^−^	+	−	+
*M*. *fructicola* ^T^				
11‐1456	lys^−^ pro^−^	(+)	−	(+)
11‐1595	aux	−	−	+
*M*. *shanxiensis* ^T^				
11‐1605	asp^−^	+	−	+
11‐1620	his^−^	−	−	−?
*M*. *sinensis* ^T^				
11‐1609	ade^−^	(+)	−	+
11‐1610	ade^−^	−	−	+
*M*. *zizyphicola* ^T^				
11‐1604	asp^−^	+	−	+
11‐1621	lys^−^	+	−	+

Strain^a^	Marker	*M*. *andauensis* ^T^	
		11‐1598 arg^−^	11‐1599 try^−^
*M*. *andauensis* ^T^			
11‐1598	arg^−^	−	+
11‐1599	try^−^	+	−

Abbreviations: +, continuous growth at intersections; (+), few individual colonies at intersections; −, no growth at intersections.

^a^ T, type strain.

## 4 DISCUSSION

Previous studies revealed that ambiguous nucleotides (SNP sites) in the ITS and D1/D2 sequences of *Metschnikowia andauensis* and *M*. *fructicola* type strains obtained by Sanger sequencing (Kurtzman & Droby, 2001; Molnar & Prillinger, 2005) were the reflection of high intragenomic diversity of rDNA repeats (Sipiczki et al., 2013, 2018). Cloning and sequencing of segments of individual repeats further showed that the strains shared a continuous pool of repeats. When subjected to phylogenetic analyses, no barcode gaps suitable for differentiation of the species were seen on the trees. In this study, the analysis was extended to all species of the *pulcherrima* clade of the genus *Metschnikowia*. First, a blast search was performed to identify all sequences in the INSDC databases which showed at least 95% identity with the D1/D2 or the ITS1–5.8S–ITS2 sequences (“taxonomic sequences”) used for the delimitation of the species of the clade. On the trees inferred from the downloaded database sequences no clear clusters were formed around the type strains. Besides, numerous sequences contained ambiguous nucleotides, and the sequences deposited for certain type strains by different authors were different. The latter findings indicated that intragenomic rDNA heterogeneity was not confined to the above‐mentioned two type strains.

To examine the possibility that other strains may also have heterogeneous rDNA, ITS and D1/D2 sequences were cloned from a further four type strains of the clade. As surmised, high intragenomic heterogeneity was found in all of them, even in those (the *M*. *shanxiensis*, *M*. *sinensis* and *M*. *zizyphicola* type strains) for which unambiguous sequences were deposited in the databases. The reason for the incongruence between the unambiguous “taxonomic sequences” of the latter strains and the revealed intragenomic diversity of their rDNA segments is that the former themselves were cloned sequences (Xue et al., 2006) representing only single repeats. Consistent with the cloning results, heterogeneous rDNA repeats were also found in the whole‐genome sequences. Interestingly, the taxonomic sequences on which the delimitations of the species were based were not found among the clones. The reason for this is that the taxonomic sequences may not be existing sequences but majority‐rule consensus sequences generated by the algorithm used for processing of the data produced by the sequencers. Because of the high intragenomic heterogeneity, different laboratories using different protocols can produce different taxonomic rDNA sequences for the same strain. Thus, neither rDNA barcode is suitable for clear differentiation of species within the clade.

When rDNA barcoding fails to provide unambiguous results, or the results need to be verified, multilocus sequence analysis (MLSA; Mallo & Posada, 2016) can be applied. This approach uses as secondary barcodes internal segments of multiple housekeeping genes. Comparison of the *ACT1* sequences available in databases or generated in this study showed that the *ACT1* genes cannot be used for species differentiation because their internal segments used for barcoding are identical or almost identical in the type strains. The barcode segments of *TEF1*, *EF2* and *RPB2* are diverse across the type strains but their Sanger sequences contain ambiguous nucleotides, indicating that the strains have two or more different copies of these genes. As shown by the comparison of the cloned segments and the genes found in the sequenced genomes, their intragenomic diversity is comparable with or even higher than the interstrain diversity. Hence, the non‐rDNA barcodes cannot be used for differentiation of the species of the *pulcherrima* clade either.

Taking all the barcode data together, it can be concluded that although the range of barcode diversity is broad enough (except for *ACT1*) for the differentiation of all known species of the clade, barcode gaps required for this are not formed due to the very high intrastrain (intragenomic) differences (Figure 2).

Examination of the sequenced genomes suggests that the internal barcode diversity might be attributed to chimeric (admixed) genome structures. The genomes of the sequenced strains, including three type strains, are heterogeneous in size and in the copy number of genes, and have high numbers of SNPs (ASM1490579v1; ASM974605v1; ASM993245v1; Gore‐Lloyd et al., 2019; Piombo et al., 2018; Venkatesh et al., 2018). The analysis performed in this study revealed that even the smallest genome (APC 1.2) assembled in a set of single‐copy chromosomes has certain genes in two copies. The analysis also showed that the di‐ and multicopy genes differed from each other in all genomes and were frequently more similar to their counterparts in other genomes. These results show that they were not paralogues but orthologues of different evolutionary histories. Thus, the sequenced strains had chimeric genomes consisting of mosaics of different origins.

How can chimeric genomes arise? Examination of stabilized chimeric (“hybrid”) strains and laboratory‐made synthetic *Saccharomyces* and *Zygosaccharomyces* hybrids revealed that chimeric genomes can arise via postzygotic evolution of hybrid genomes (e.g., Chand Dakal et al., 2016; Sipiczki, 2018). The postzygotic changes can reduce the ploidy (descending dysploidy) by eliminating large parts of the parental subgenomes and allow interactions between them by recombination. The evolving (stabilizing) hybrid only retains admixed parental mosaics (haplotype segments). Analogous processes can be assumed to take place in the *pulcherrima* clade. In a previous study, the type strains of *M*. *andauensis* and *M*. *fructicola* were found to form viable hybrids with the type strain of *M*. *pulcherrima* (Sipiczki et al., 2018). The mating tests performed in this study revealed that *M*. *shanxiensis*, *M*. *sinensis* and *M*. *zizyphicola* ex‐type strains also can hybridize with the *M*. *pulcherrima* ex‐type strain. Natural hybridization can take place in mixed populations colonizing various natural substrates or in guts of fruit‐visiting insects as proposed for *Saccharomyces* (Stefanini et al., 2016). The viability of the *pulcherrima*‐clade hybrids implies that the type strains are not isolated by prezygotic reproductive barriers. To investigate the possibility that they are postzygotically isolated (by hybrid sterility), their hybrids were tested for the production of gametes, which are ascospores in ascomycetous yeasts. Most of the hybrids produced only very few asci and the ascospores did not germinate. The poor sporulation can be attributed to the collapse of meiosis caused by promiscuous pairing of chromosomes or segments of chromosomes of different origins and structures as shown in *Saccharomyces* (e.g., Lorenz et al., 2002). Thus, the type strains seem to be reproductively isolated by a postzygotic sterility barrier. However, in spite of the sterility of the hybrids, segregation can take place. When cultivated in nonselective conditions, segregants with parental phenotypic traits appeared in the hybrid cultures most probably due to a process similar to GARMi, a gradual genome reduction process observed in *Saccharomyces* hybrids that can result in chimeric genomes (Sipiczki, 2018). This observation is consistent with the findings of Pitt and Miller (1970), which indicated that *M*. *pulcherrima* has a parasexual life cycle.

In a genome of chimeric composition, rDNA repeats can occur in mosaics of different origins and thus the genome may have several short tandem arrays or dispersed small groups of repeats or even singletons. With scattered locations of their repeats, the *pulcherrima*‐clade species represent rare exceptions among fungi. When the repeats are dispersed, they cannot be efficiently homogenized because the homogenization process that keeps the rDNA repeats highly uniform in other organisms requires continuous rDNA arrays (for a review, see Eickbush & Eickbush, 2007). When the repeats cannot be (efficiently) homogenized, the organism has to secure the sufficient number of functional genes in different ways (Eirín‐López et al., 2012). In a previous study, the birth‐and‐death mechanism was proposed to substitute the homogenization process in *M*. *fructicola* (Sipiczki et al., 2018). The results of the current study suggest that the same mechanism may operate in the other type strains as well. The birth‐and‐death process cannot standardize the repeat sequences, and thus multiple functional repeat variants differing at neutral positions can coexist in the genome. In the reconstructed secondary structures of the transcripts of the cloned and genomic repeats, the variable (di‐ and polymorphic) sites located in stems of loops were wobble parings or compensatory mutations in the complementary pairing segment of the stem that could neutralise the effect of the substitutions on the structure of the RNA transcript. During the course of evolution, the structure of a given RNA can be maintained via CBCs that occur among covarying nucleotides in paired regions (for a review, see Rivas, 2021). The high diversity of the structures hints that some of them may not be fully active. The truncated repeats detected previously in the *M*. *fructicola* genome (Sipiczki et al., 2018) and in this study in other genome sequences may be worn‐out repeats being gradually degraded in the “death” part of the birth‐and‐death process. The assumption that there is a causative relationship between the dispersed location of rDNA units and their intragenomic diversity in the *Metschnikowia* genomes is consistent with the observed correlation between the number of ITS ribotypes and the number of locations of rDNA units in the genomes of *Nicotiana* species (Matyasek et al., 2012). Nevertheless, birth‐and‐death evolution and concerted evolution may operate simultaneously, as has been proposed for the 5S rDNA (Freire et al., 2010).

However, the high rDNA diversity cannot be attributed solely to the lack of homogenization. The phylogenetic analysis failed to group the cloned and genomic sequences of the *pulcherrima*‐clade type strains in distinct clusters and the sequences of the type strains were located intermixed. The latter finding hinted at “interstrain” interactions of the rDNA repeats of the strains. A similar conclusion could be drawn from the comparison of the protein‐coding genes. When a gene was present in two or more copies in a genome, its copies were frequently less similar to each other than to their counterparts in other strains, which could be interpreted as having discordant evolutionary histories (derived from different ancestors). Mixing of evolutionary histories is referred to as reticulation, a network‐like evolutionary process in which genes and genomes (lineages) can evolve through interchanging genetic material, causing branches in an evolutionary tree to merge, that is forming a net (or network) instead of a purely (strictly) bifurcating tree (for a review, see Swithers et al., 2012). Network analysis of the ITS and D1/D2 LSU sequences cloned from the ex‐type strains or identified in the sequenced genomes clearly demonstrates that reticulation did indeed take place in the evolution of the clade.

Recent genomic analyses are showing that reticulation is much more widespread in evolution than previously thought (for a review, see, e.g., Mallet et al., 2016). For example, reticulate species complexes are common in plants (e.g., Burnier et al., 2009; Nauheimer et al., 2019; Sandstedt et al., 2020). Reticulation leads to heterozygosity of large portions of the genome (e.g., segmental di‐ or polyploidy) and/or mosaic (chimeric) genome structures. Mosaic (chimerized, admixed) genomes have been found in many groups of bacteria (for a review, see Martin, 1999), plants (recent examples: Ahmed et al., 2019; Baurens et al., 2019; Curk et al., 2014; Sandstedt et al., 2020) and animals (e.g., Kim et al., 2020; Yang et al., 2021). However, this study is the first attempt to find correlation between intragenomic barcode diversity and the chimeric structures of genomes.

Relying on a few standard barcodes and neglecting the admixed origin of the mosaics in the chimeric genomes can easily mislead the analysis. Thus, reconstruction of the evolutionary history of such genomes and identification of their positions in the taxonomic system of yeasts will need a more complex approach than the use of only a few barcode sequences representing only small fractions of the genomes. In particular, this is the case when no pure ancient genomes are known (if they exist at all). Two previous studies focused on rDNA diversity in the *M*. *andauensis* and *M*. *fructicola* type strains raised the question of whether it is appropriate to maintain the species status of these organisms given the lack of a separating barcode gap between them (Sipiczki et al., 2013, 2018). Based on the results presented here, the question can be extended to the whole clade. Detailed taxonomic examinations (including “conventional taxonomic tests,” phenotypic comparisons and MLSA) on larger groups of strains will need to be carried out to answer the question. Genome chimerization and intragenomic barcode diversity can cause incorrect identifications in metabarcoding studies and result in overestimation of species richness. A possible solution to the problem caused by intragenomic barcode diversity could be the use of “consensus” barcode sequences with di‐, tri‐ or tetramorphic (“polymorphic”) sites in the positions where two, three or four nucleotides alternate.

## CONFLICT OF INTEREST

The author declares no conflict of interest.

## BENEFIT‐SHARING STATEMENT

There are no benefits to report.

## Supporting information

Supplementary Material

## Data Availability

The sequences generated in this study are deposited in GenBank under registration numbers listed in Table S1. All trees shown are also available in TreBASE: 28355, 28358, 28359, 28405.
