# Supplementary material for: When barcoding fails: Genome chimerization (admixing) and reticulation obscure phylogenetic and taxonomic relationships
Source: Mol Ecol Resour. 2022 Feb 3;22(5):1762–85. doi: 10.1111/1755-0998.13586 (PMC9303175; doi:10.1111/1755-0998.13586)
Supplement: Supplementary file 1 — Supplementary Material [file MEN-22-1762-s001.pdf]

MOLECULAR ECOLOGY RESOURCES

Supplemental Information for:

When barcoding fails: genome chimerisation (admixing) and reticulation obscure phylogenetic and taxonomic relationships.

M. Sipiczki

Table of Contents:

Methods	Page 1
TABLE S1	Page 9
FIGURE S1	Page 10
FIGURE S2	Page 11
FIGURE S3	Page 12
FIGURE S4	Page 13

METHODS

1. Culture media

The composition of the growth media YEA (yeast-extract agar), YEL (yeast-extract liquid), and SMA (synthetic minimal agar) were described previously (Sipiczki, 2012). To visualise differences in pulcherrimin production, the medium was supplemented with 0.2 mg/ml $FeCl_3$. Sporulation was tested on vegetable juice (BIO Gemüsesaft, Josef Pölz, Bio-Produkte, 84518 Garching an der Alz, Germany; 20-fold dilution) agar (Sipiczki, Horvath, & Pfliegler, 2018).

2. DNA isolation, PCR amplification and cloning

DNA was isolated from overnight cultures grown in YEL and the isolated DNA was used for the amplification of barcode sequences using GoTaq polymerase (Promega, Madison, United

MOLECULAR ECOLOGY RESOURCES

States). The D1/D2 domains of the LSU rRNA genes and ITS1-5.8S-ITS2 segments of the rDNA repeats were amplified with the primer pairs NL1-Nl4 (O'Donell, 1993) and ITS1-ITS4 (White, Bruns, Lee, & Taylor, 1990), respectively. The primers used for the amplification of barcode segments of the protein-encoding genes were CA16mod and CA5R for *ACT1* (Molnar & Prillinger, 2005), YTEF-1 (5'-GGTCAYGTYGAYKCTGGTAAGT) and YTEF-6A (5'-GGTABTCRSTGAARGYYTCAACRGACA) for *TEF1* (Kurtzman & Robnett, 2003), EFIIF1 (AAGTCTCCAAACAAAGCATAAC) and EFIIR2 (GGGAAAGCTTGACCACCAGTAGC) for *EF2* (Diezmann, Cox, Schönian, Vilgalys, & Mitchell, 2004), RPB2-6F and fRPB2-7CR for *RPB2* (Kurtzman & Robnett, 2003). The PCR products were used for direct sequencing in both directions with the same primers or for random cloning of fragments into the pGEM-T Easy Vector (Promega, Madison, United States), following the manufacturer's instructions. Upon transformation of *E. coli* cells with the plasmids, bacterial colonies were isolated randomly and plasmids were extracted from the isolates. The inserts of the plasmids were sequenced in both directions with the primers pairs used for amplification. The sequences were deposited in GenBank under accession numbers listed in Supporting Information Table S1.

3 Identification of sequences in the INSDC databases and in *Metschnikowia* genome sequences

Search for barcode sequences in the INSDC databases was performed using the Blastn service of NCBI (https://blast.ncbi.nlm.nih.gov/Blast.cgi) and the sequences of the type strains of the species as query sequences. Search for sequences in whole-genome sequences was performed

in the Genome database of NCBI (https://www.ncbi.nlm.nih.gov/genome/). The database barcode sequences of the type strains were used as query sequences to identify the corresponding genes. For the identification of other (non-barcode) genes, sequences of the annotated *M.* aff. *pulcherrima* APC1.2 genome were used. However, this genome has no “obvious” *MATalpha1* gene. To search the genome sequences for this gene, the *Clavispora lusitaniae* orthologues (CLUG_04923) of the *S. cerevisiae* genes *MATalpha1* (YCR040W) were used.

4. **Phylogenetic and network analyses**

For phylogenetic and network analysis the D1/D2 and ITS sequences overlapping with a 499-nt long D1/D2 and a 354-nt long ITS1-5.8S-ITS2 segment of the *M. pulcherrima* CBS 5833^T database sequences **U45736 and** JX188180 were used. Multiple alignments were obtained with the Clustal W 1.7 (Thompson, Higgions, & Gibson, 1994), and the MUSCLE 3.8.31 (Edgar, 2004) algorithms. *Trees were* generated from the alignments using *neighbor*-joining and maximum-likelihood analysis. In the neighbor-joining analysis (Phylip 3.67 software package; Felsenstein, 2007) the F84 model of nucleotide substitution (Felsenstein & Churchill, 1996) was used for computing distance matrices. Confidence limits were estimated from bootstrap analysis based on 1000 replication. The maximum-likelihood trees were generated with the PhyML 3.0 algorithm (Guindon et al., 2010) using the best model suggested by the Akaike Information Criterion (AIC) in jMedelTest version 1.0.2 (Posada, 2008). In all analyses, the D1/D2 (AY452039) or the ITS (AY494780) sequence of *Candida picachoensis* (*M. picachoensis*) CBS 9804^T were used

MOLECULAR ECOLOGY RESOURCES

as outgroups. This species is close enough to the *pulcherrima* clade (Lachance, 2011) to have sequences moderately related to the analysed sequences but far enough to be an uncontroversial outgroup. Trees were visualised with the FigTree programme (http://tree.bio.ed.ac.uk/software/figtree/). Circular (polar) layout was chosen and the tip labels were aligned to the circumference of the circle. From the sequence alignments neighbor-net splits graphs were also created with the SplitsTree4 V4.17.0 package (Huson & Bryant, 2006). Statistical parsimony networks were constructed with TCE 1.21, a program that implements the estimation of genealogies of DNA sequences from their multiple alignments (Clement et al., 2000). The default 95% cutoff was used.

5. **RNA secondary structure prediction**

Minimum free energy secondary structures were generated for the D1/D2 loops with **Predict, a secondary structure webserver** **(**http://rna.urmc.rochester.edu/RNAstructureWeb/Servers/Predict1/Predict1.html**)** for the hairpin loops **using default settings.** Minimum free-energy secondary ITS1 structures were predicted with the algorithm available at the RNAfold Webserver (http://rna.tbi.univie.ac.at/cgi-bin/RNAWebSuite/RNAfold.cgi) (Lorenz et al., 2011). For the ITS2 secondary structure prediction, the ITS2 database pipeline: (Ankenbrand, Keller, Wolf, Schultz, & Förster, 2015) available at http://its2.bioapps.biozentrum.uni-wuerzburg.de/ was used. The ITS secondary structures were re-drawn for publication purposes using the R-chie webserver

https://e-rna.org/r-chie/ (Lai, Proctor, Zhu, & Meyer, 2012) and the forna package http://rna.tbi.univie.ac.at/forna/ (Kerpedjiev, Hammer, & Hofacker, 2015).

6. Testing of sporulation, spore viability and mitotic segregation

Sporulation was examined microscopically in cultures grown on plates of vegetable juice (20-fold dilution) agar after 4 weeks of incubation at 17°C. For the examination of spore germination, samples of suspensions of sporulating cultures were spread on thin YEA films prepared on glass slides and covered with cover slips. These sandwich cultures were incubated at 20 °C and examined microscopically at regular time intervals for one week. For the detection of mitotic segregation, hybrids of mutants differing in the intensity of pulcherrimin production were streaked on YEA medium supplemented with 0.02 mg/ml $FeCl_3$. Segregants appeared as sectors of different pigmentation intensities at the edge of the colony.

References

Ankenbrand, M. J., Keller, A., Wolf, M., Schultz, J., & Förster. F. (2015). ITS2 Database V: Twice as much. *Molecular Biology and Evolution,* 32, 3030-3032. doi: 10.1093/molbev/msv174

Clement, M., Posada, D., & Crandall, K. A. (2000). TCS: a computer program to estimate gene genealogies. *Molecular Ecology*, 9, 1657–1660. doi: 10.1046/j.1365-294x.2000.01020.x

Diezmann, S., Cox, C. J., Schönian, G., Vilgalys, R. J., & Mitchell, T. G. (2004). Phylogeny and evolution of medical species of Candida and related taxa: a multigenic analysis. *Journal of Clinical Microbiology,* 42, 5624-5635. doi: 10.1128/JCM.42.12.5624-5635.2004

Edgar, R. C. (2004). MUSCLE: multiple sequence alignment with high accuracy and high throughput. *Nucleic Acids Research,* **32**: 1792-1797. DOI: 10.1093/nar/gkh340

Felsenstein, J. (2007). PHYLIP (phylogeny inference package), version 3.67. Distributed by the author. Department of Genome Sciences, University of Washington, Seattle, USA.

Felsenstein, J., & Churchill, G. A. (1996). A Hidden Markov Model approach to variation among sites in rate of evolution. *Molecular Biology and Evolution,* 13, 93–104. doi: 10.1093/oxfordjournals.molbev.a025575

Guindon, S., Dufayard, J. F., Lefort, V., Anisimova, M., Hordijk, W., & Gascuel, O. (2010). New algorithms and methods to estimate maximum-likelihood phylogenies: Assessing the performance of PhyML 3.0. *Systematic Biology,* 59, 307–321. doi: 10.1093/sysbio/syq010

Huson, D. H., & Bryant, D. (2006). Application of phylogenetic networks in evolutionary studies, *Molecular Biology and Evolution,* 23, 254-267. doi: 10.1093/molbev/msj030

Kerpedjiev, P., Hammer, S., & Hofacker, I. L. (2015). Forna (force-directed RNA): Simple and effective online RNA secondary structure diagrams. *Bioinformatics,* 31, 3377-3379. doi:10.1093/bioinformatics/btv372

Kurtzman, C. P., & Robnett, C. J. (2003). Phylogenetic relationships among yeasts of the '*Saccharomyces* complex' determined from multigene sequence analyses. *FEMS Yeast Research,* 3, 417-432. doi: 10.1016/S1567-1356(03)00012-6

Lachance, M.-A. (2011). *Metschnikowia* Kamienski (1899). In C. P. Kurtzman, J. W. Fell, & T. Boekhout (Eds.), *The yeasts. A taxonomic study* (pp. 575-620). Amsterdam, the Netherlands: Elsevier. doi: 10.1016/B978-0-444-52149-1.00046-X

Lai, D., Proctor, J. R., Zhu, J. Y., & Meyer, I. M. (2012). R-CHIE: a web server and R package for visualizing RNA secondary structures. *Nucleic Acids Research,* 40, e95. doi: 10.1093/nar/gks241

Lorenz, R., Bernhart, S. H., Höner zu Siederdissen, C., Tafer, H., Flamm, C., Stadler, P. F., & Hofacker, I. L. (2011). ViennaRNA Package 2.0. *Algorithms for Molecular Biology,* 6, 26. doi: 10.1186/1748-7188-6-26

Molnar, O., & Prillinger, H. (2005). Analysis of yeast isolates related to *Metschnikowia pulcherrima* using the partial sequences of the large subunit rDNA and the actin gene; description of *Metschnikowia andauensis* sp. nov. *Systematic and Applied Microbiology,* 28, 717–726. doi: 10.1016/j.syapm.2005.05.009

O'Donnell, K. (1993). *Fusarium* and its near relatives. In D. R. Reynolds, & J. W. Taylor (Eds.), *The fungal holomorph: mitotic, meiotic and pleomorphic speciation in fungal systematics* (225-233). Wallingford, UK: CAB International. ISBN: 0851988652

Posada, D. (2008). jModelTest: Phylogenetic Model Averaging. *Molecular Biology and Evolution,* 25, 1253–1256. doi: 10.1093/molbev/msn083

Sipiczki, M. (2012). *Pichia bruneiensis* sp. nov., a biofilm-producing dimorphic yeast species isolated from flowers in Borneo. *International Journal of Systematic and Evolutionary Microbiology,* 62, 3099-3104. doi: 10.1099/ijs.0.044974-0

Sipiczki, M., Horvath, E., & Pfliegler, W. P. (2018). Birth-and-death evolution and reticulation of ITS segments of *Metschnikowia andauensis* and *Metschnikowia fructicola* rDNA repeats. *Frontiers in Microbiology,* 9, 1193. doi: 10.3389/fmicb.2018.01193

Thompson, J. D., Higgions, D. G., & Gibson, T. J. (1994). CLUSTALW: improving the sensitivity of progressive multiple sequence alignment through sequence weighting, position-specific gap penalties and weight matrix choice. *Nucleic Acids Research,* 22, 4673–4680. doi: 10.1093/nar/22.22.4673

White, T. J., Bruns, T., Lee, S., & Taylor, J. (1990). Amplification and sequencing of fungal ribosomal RNA genes for phylogenetics. In M. A. Innis, D. H. Gelfand, J. J. Sninsky, & T. J. White (Eds.), *PCR protocols. A guide to methods and applications* (pp. 315-322). San Diego, CA: Academic Press.

MOLECULAR ECOLOGY RESOURCES

TABLE S1. Accession numbers of cloned rDNA sequences.

Segment	*M. andauensis* 11-1120 (CBS 10809^T)		*M. fructicola* 11-579 (CBS 8853^T)		*M. pulcherrima* 11-578 (CBS 5833^T)		*M. shanxiensis* 11-1090 (CBS 10359^T)		*M. sinensis* 11-1088 (CBS 10357^T)		*M. zizyphicola* 11-1089 (CBS 10358^T)	
	Clone	Accession number	Clone	Accession number	Clone	Accession number	Clone	Accession number	Clone	Accession number	Clone	Accession number
ITS	Ian42	KM243743	Ifr4	KM213977	Ipua52	KM209321	Ish133411	KM243731	Isi13321	KM243713	Izi13337	KM243722
	Ian43	KM243744	Ifr6	KM213978	Ipub65	KM209322	Ish133412	KM243732	Isi13322	KM243714	Izi13338	KM243723
	Ian51	KM243745	Ifr9	KM213979	Ipub69	KM209323	Ish133413	KM243733	Isi13324	KM243715	Izi13339	KM243724
	Ian5	KM243746	Ifrb13	KM213980	Ipub71	KM209324	Ish1337a	KM243734	Isi13351	KM243716	Izi1336a	KM243725
	Ian6	KM243747	Ifrb15	KM213981	Ipuc3	KM209325	Ish1337e	KM243735	Isi13354	KM243717	Izi1336f	KM243726
	Ian10	KM243748	Ifrb17	KM213982	Ipuc6	KM209326	Ish1337g	KM243736	Isi13358	KM243718	Izi1336h	KM243727
			Ifrc26	KM213983	Ipuc10	KM209327	Ish13372/2	KM243737	Isi13351/1	KM243719	Izi13364/b	KM243728
			Ifrc27	KM213984			Ish13372/3	KM243738	Isi13351/3	KM243720	Izi13364/c	KM243729
							Ish13372/4	KM243739	Isi13351/5	KM243721	Izi13364/e	KM243730
							Ish13375/2	KM243740				
							Ish13375/c	KM243741				
							Ish13375/d	KM243742				
D1/D2	a77	KC411953	fb1	KC411962	1mut8	KM249361	d8h14	KM350705	d6f2	KM275352	d7g6	KM275366
	a78	KC411954	fb3	KC411963	1mut9	KM249362	d8h16	KM350706	d6f3	KM275353	d7g7	KM275367
	aa20	KC411955	fb6	KC411964	1mut10	KM249363	b2	KM350707	d6f4	KM275354	d7g8	KM275368
	aa23	KC411956	fb9	KC411965	2mut15	KM249364	d2	KM350708	b4	KM275355	a15	KM275369
	aa23a	KC411957	fb10	KC411966	2mut17	KM249365	d3	KM350709	b10	KM275356	a17	KM275370
	ab24	KC411958	fb11	KC411967	a37	KM249366	d4	KM350710	e2	KM275357	a18	KM275371
	ab27	KC411959	fc15	KC411968	a42	KM249367	a	KM350711	e3	KM275358	22a	KM275372
			fc17	KC411969	a46	KM249368	c	KM350712	e4	KM275359	22b	KM275373
			fc21	KC411970	b51	KM249369	f	KM350713	f9	KM275360	22f	KM275374
			f39a1	KC411960	b54	KM249370			f10	KM275361		
			f39b2	KC411961	b58	KM249371			f11	KM275362		
					c61	KM249372			21-1	KM275363		
					c62	KM249373			21-4	KM275364		
					c64	KM249374			21-5	KM275365		

MOLECULAR ECOLOGY RESOURCES

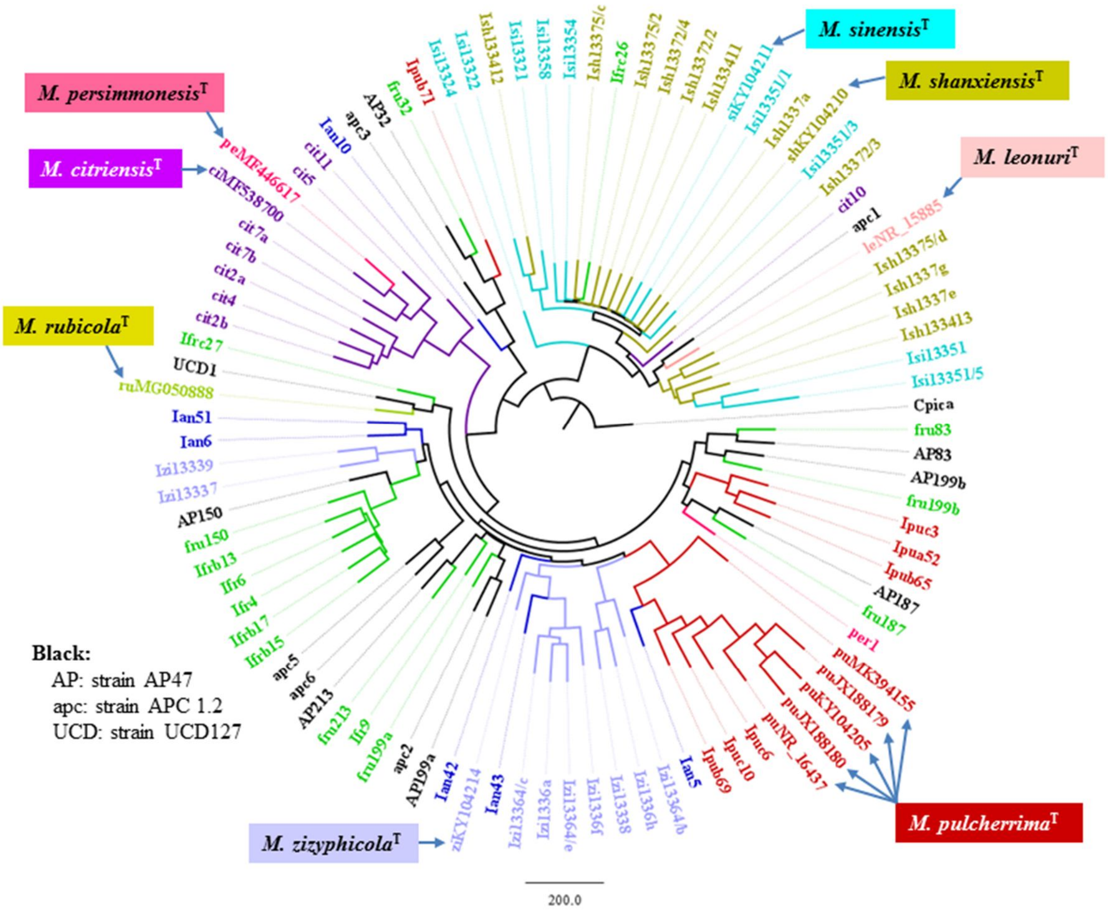

FIGURE S1 A tree derived from the neighbour-joining analysis of the ITS1-5.8S-ITS2 sequences. The flagged sequences are „taxonomic" sequences on which the delimitation of the species was based. The rest is cloned from the type strains or found in the genome sequences. Outgroup: *Metschnikowia* (*Candida*) *picachoensis* CBS 9804^{T} (AY494780). Each type strain is shown in a different colour. Black: genomes of non-type strains.

MOLECULAR ECOLOGY
RESOURCES

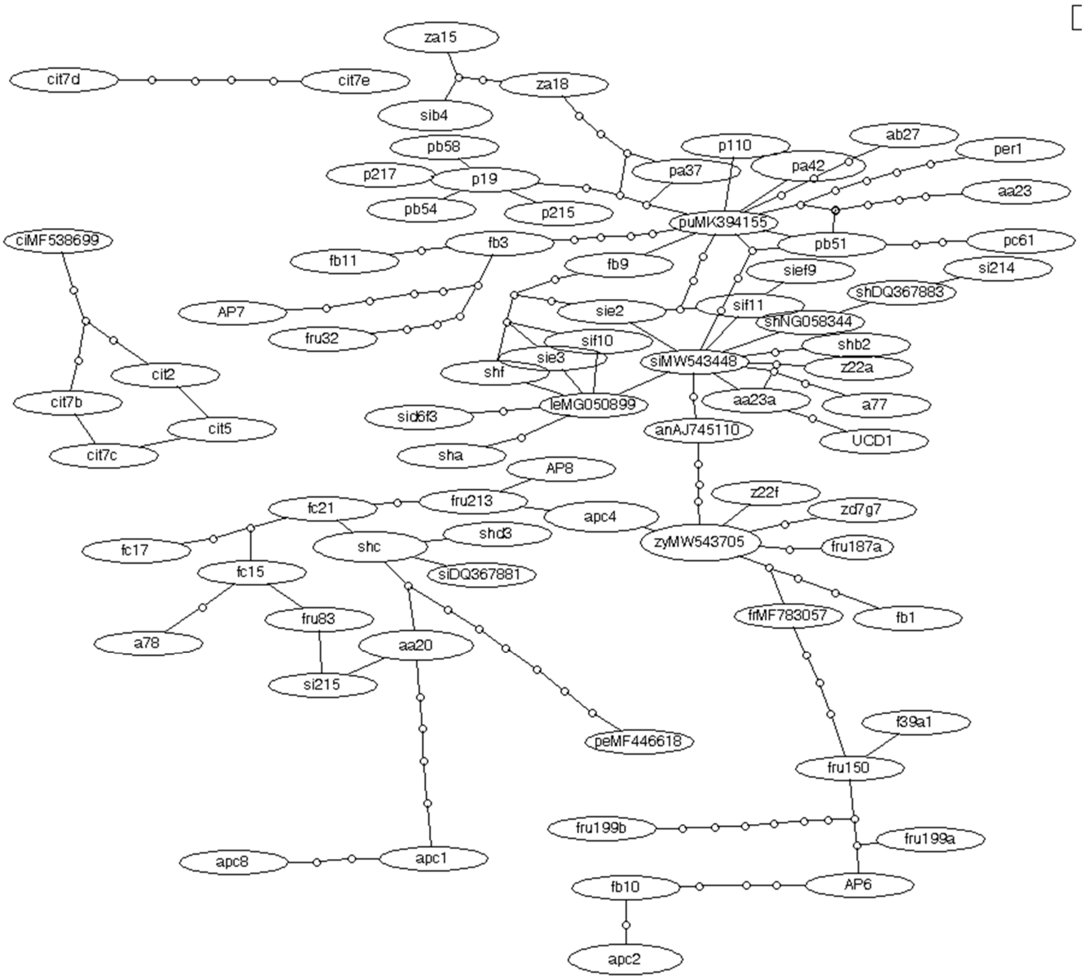

FIGURE S2. Parsimony network of of all cloned and genomic D1/D2 sequences. For the origin of the sequences, see the colour codes in Fig. 7.

MOLECULAR ECOLOGY
RESOURCES

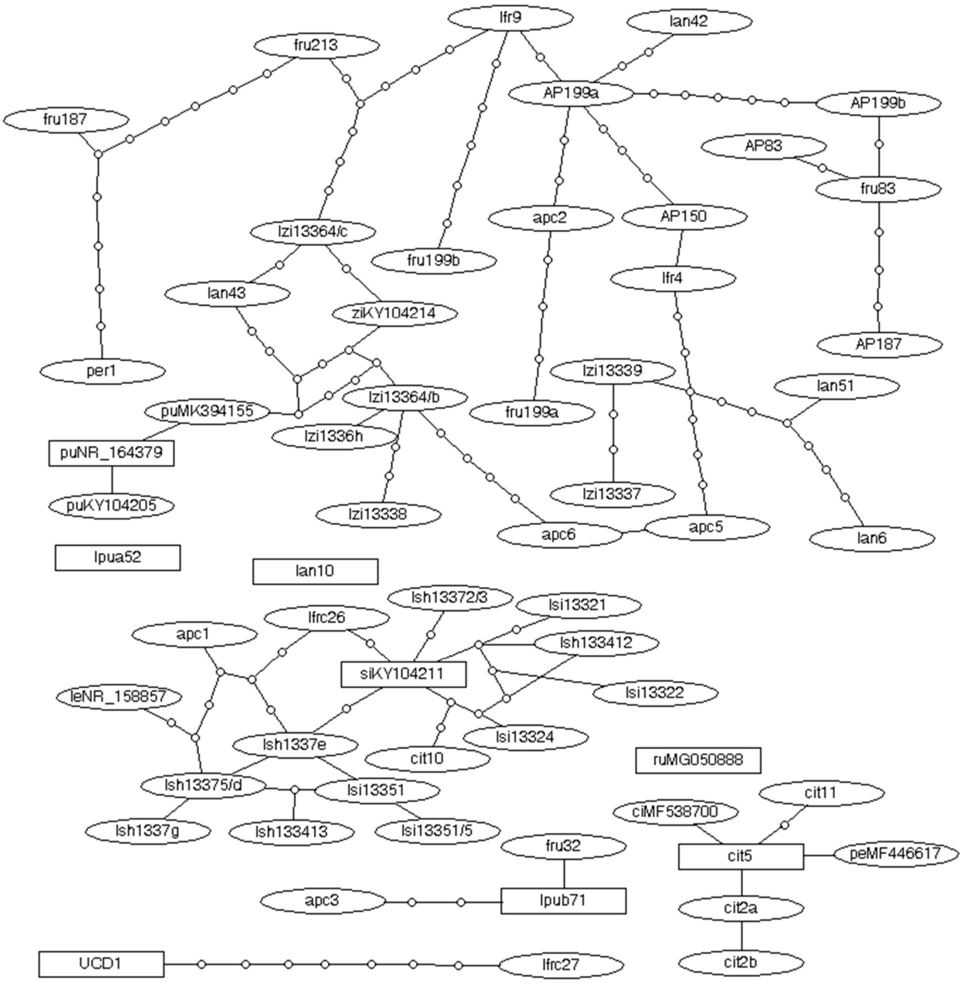

FIGURE S3. Parsimony network of of all cloned and genomic ITS sequences. For the origin of the sequences, see the colour codes in Fig. 6.

MOLECULAR ECOLOGY RESOURCES

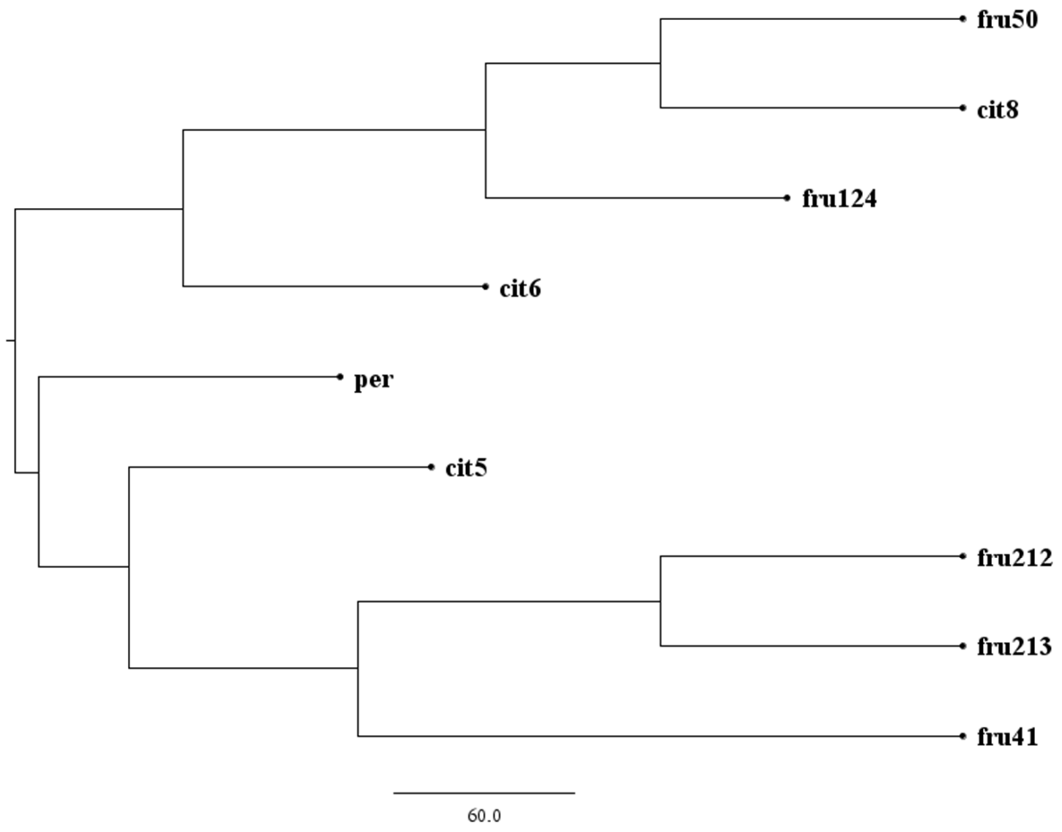

FIGURE S4. Neighbor-join tree of *TEF1* genes of type-strain genomes. cit5, cit6, and cit8: *M. citriensis* CICC33213^{T} contigs 5, 6 and 8, respectively. fru41, fru50, fru124, fru212, and fru213: *M. fructicola* CBS 8853^{T} unitigs 41, 50, 124, 212 and 213, respectively. per: *M. persimmonesis* KIOMG15050^{T}.
